# Cravings, Control, and Cessation: A Scoping Review of Perceptions of Nicotine Addiction

**DOI:** 10.1007/s40429-025-00673-4

**Published:** 2025-07-11

**Authors:** Allison A. Temourian, Deanna M. Halliday, Anna V. Song

**Affiliations:** 1 Center for Tobacco Control Research and Education, University of California, San Francisco, USA; 2 Nicotine and Cannabis Policy Center, University of California, Merced, USA; 3 Department of Psychological Sciences, School of Social Sciences, Humanities, & Arts, University of California, Merced, USA

**Keywords:** Addiction, Nicotine, Perceptions of addiction, Tobacco

## Abstract

**Purpose of Review:**

Nicotine addiction is the result of repeated tobacco use and subsequently promotes continued consumption, potentially acting as both cause and consequence of tobacco use. This scoping review aims to describe the literature and catalogue existing measures regarding perceptions of nicotine addiction with special attention to scales that recognize its multidimensionality.

**Recent Findings:**

Following a comprehensive review of 923 empirical articles, we found 252 articles that assessed perceptions of nicotine addiction, five of which utilized a validated measure. Single item assessments were categorized into affective concern, knowledge that tobacco is addictive, personal perceptions of addiction, other people’s addiction, and comparative addictiveness. Scaled measures of perceptions of nicotine addiction largely assessed perceived susceptibility and severity.

**Summary:**

Despite decades of research demonstrating the importance of perceptions of risk and expectancies in risk-behavior decision-making, tools and items assessing perceptions of nicotine addiction are highly varied and do not account for the multidimensionality of nicotine addiction. We, as a field, lack a comprehensive assessment of perceptions of nicotine addiction that integrates the complexity of addiction into an individual’s appraisal of risk, which is a critical component of prevention and intervention-based research.

**Supplementary Information:**

The online version contains supplementary material available at 10.1007/s40429-025-00673-4.

## Introduction

Tobacco remains the leading cause of preventable death with an annual mortality rate of eight million people worldwide [1]. In 2021, roughly 23.6% of United States (U.S.) adults (aged 18 years +) were past month tobacco users (including people who vape) [2]. Compared to other addictive substances (e.g., heroin, methamphetamines), epidemiologically, one of the most common addictions is tobacco [3–6]. Prevalence of nicotine addiction in the U.S. remains problematic, with many people – including those with diagnosed substance use disorder (i.e., experiencing addiction) – unsuccessfully attempting to quit using tobacco [7].

### Nicotine Addiction

Nicotine is the addictive component of tobacco products that sustains tobacco use [8, 9]. People can ingest nicotine through modalities such as cigarettes, electronic cigarettes (e-cigarettes), and other forms. Using any form of tobacco is not without risk, thus, it is important to understand the duality of nicotine addiction: 1) addiction contributes to a host of negative health outcomes related to tobacco use including, but not limited to, chronic obstructive pulmonary disease and respiratory tract infections, among others [10], and 2) addiction is an outcome/disorder itself that is associated with social, physical, emotional, and psychological consequences.

Typically, research tends to focus on the consequences of addiction. For example, the National Institute on Drug Abuse (NIDA) [11] defines addiction as a, “chronic, relapsing disorder characterized by compulsive drug seeking, continued use despite harmful consequences, and long-lasting changes in the brain.” Similarly, the definition of substance use disorder in the Diagnostic and Statistical Manual of Mental Disorders 5th edition (DSM-5) [12] roughly corresponds to NIDA’s use of the term “addiction” (note: the DSM-5 does not use the term “addiction”) [13]. Whereas experts in the field have substituted the term “addiction” with a more robust term like *dependence*, lay individuals discussing habitual use of nicotine products colloquialize this with the former term [14, 15]. In this review, we utilize the term more commonly used by the general public when referring to being unable or unwilling to stop using tobacco/nicotine when it is in a person’s best interest to do so [16].

### Importance of Understanding Nicotine Addiction

Despite the field’s operational definition of addiction and the ability to identify addiction, this may not translate to the general public. Specifically, even though researchers and clinicians have identified important characteristics and consequences of addiction, lay people may have a different concept and understanding of addiction. A discrepancy between scientific and lay understanding of addiction is important, as theory and empirical work have consistently demonstrated that perceptions and understanding of risks and consequences strongly relate to health behaviors [17, 18].

Several theories, including the Common Sense Model [19], Theory of Planned Behavior [20], Health Belief Model [21], and the Social Cognitive Model [22], posit that perceptions are critical in understanding health behaviors and outcomes; furthermore, the negative consequences of these health behaviors remain undesirable and typically avoided. The Common Sense Model assesses a host of illness representations including perceived consequences, personal control, and risk appraisal. In a tobacco use context, this model has mainly been used to understand the health consequences of tobacco use related to other smoking related illnesses, not nicotine addiction [23]. The Theory of Planned Behavior, Health Belief Model, and the Social Cognitive Model focus on perceived susceptibility, with some attention dedicated to perceived severity, perceived benefits, perceived barriers, and self-efficacy. Most of these theories and models would suggest that if people perceived nicotine addiction as harmful or as a negative consequence, they would avoid nicotine addiction by not using tobacco.

### Perceptions of Addiction in Tobacco Research

Although theories point to the importance of risk perceptions in health behavior decision making, most research endeavors focus on capturing the behavior itself, such as asking how soon after waking one uses a tobacco product. Additionally, some studies prioritize understanding the perceived addictiveness of a particular tobacco product (e.g., cigarettes, e-cigarettes) instead of their addictive component (i.e., nicotine), which may help elucidate why lay individuals appraise the addictiveness of tobacco products to varying degrees. In general, studies that may control for perceptions of nicotine addiction most commonly utilize one item, and even with one item, there is a demonstration of the relationship between perceptions of addiction and smoking-related behavior [24–27]. However, single item questions neglect to account for the multidimensionality of addiction and provide little insight into how the perceived risk of developing or worsening nicotine addiction contributes to health decision making, even though being perceived as “addicted” can be associated with a number of negative connotations that may infer problems with health, social relationships, and general well-being [14, 28]. Moreover, there is no standardized single item measure that assesses perceptions of nicotine addiction, which contributes to a lack of understanding in how people conceptualize nicotine addiction.

Clinical conceptualization of addiction, namely the DSM-5, is multidimensional. Moreover, scales used in survey research such as the Fagerström Test of Nicotine Dependence (FTND) [29] and the Nicotine Dependence Syndrome Scale (NDSS) [30] were designed to not only capture clinical criteria, but also the field’s enriched understanding of dependence and excessive use of substances. However, despite the comprehensiveness of diagnostic assessments of addiction severity/dependence, it is possible that the lay person perceives nicotine addiction using only one indicator (i.e., experiencing craving). It is also feasible that none of the indicators may reflect addiction, or that addiction is simply perceived as either “good” or “bad”. These may be addressed in the literature through assessments of perceptions of nicotine addiction.

### Inadequacies in Measurements of Perceptions of Nicotine Addiction

The misalignment of perceptions of nicotine addiction may indicate inadequate education about the nature and experiences of addiction as something to avoid. Additionally, if there is a discrepancy between scientific conceptualization of the measurement of nicotine addiction and lay people’s understanding of addiction, prevention and intervention efforts may be missing potential opportunities to promote positive health behaviors. For example, continued tobacco use despite desire to not use tobacco is a clinically recognized aspect of addiction, and roughly 70% of established tobacco users report regretting initiating in the first place [31]. If people knew that addiction was the use of a substance against their own will, it may prevent initial use and may even promote cessation. Thus, the identification of perceptions of nicotine addiction may be key not only in deterring tobacco use initiation, but also in promoting cessation.

### Aims

The purpose of this scoping review is to identify, describe, and organize assessments of perceptions of nicotine addiction within a perception of risk framework (e.g., susceptibility, severity, general risk knowledge, etc.).

## Methods

### Search Strategy

**Fig. 1:**
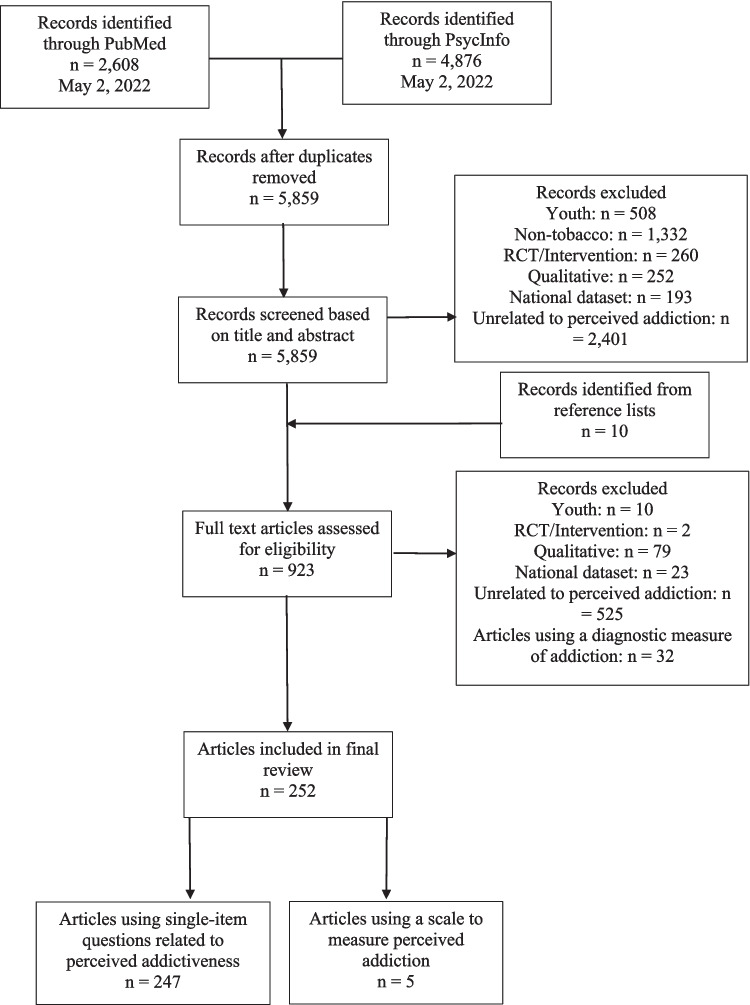
Review of Eligible Literature (PRISMA)

To identify existing papers, a review was conducted using PubMed and PsycInfo for terms relevant to three main constructs: social cognitions, substance, and addiction. The search strings for both databases can be found in Table 1. We utilized the PRISMA (Preferred Reporting Items for Systematic Reviews and Meta-Analyses) [32] reporting standards and guidelines to document the process of inclusion/exclusion and have provided a flow diagram to describe this process (Fig. 1). Reviewers used Zotero, a reference management tool, to organize papers.

**Table 1:** Database Search String

Database	Search Terms
Pubmed	(“perce*” OR “belief” OR “attitude*”) AND (“smok*” OR “tobacco” OR “nicotine”) AND (“addict*” OR “depend”)
PsycInfo	((“perce*”) OR (“belief*”) OR (“attitude*”)) AND ((“smok*”) OR (“tobacco”) OR (“nicotine”)) AND ((“addict*”) OR (“depend*”))

All titles and abstracts were screened by one reviewer and excluded if papers did not mention tobacco and/or nicotine in the title and/or abstract. After the completion of this stage, the same reviewer conducted a full-text screening with the same exclusion criteria listed below. After full-text screening was complete, the reviewer organized individual items assessing perceptions of nicotine addiction into major themes (see Table 2; Supplemental Material 1) and matched scale items into these thematic groupings as well (see Table 3). To help standardize item placement into these major themes, the reviewer developed a flowchart that answered common questions that arose during the placement process (see Supplemental Material 2). The placement of items was verified by a second reviewer.

**Table 2:**
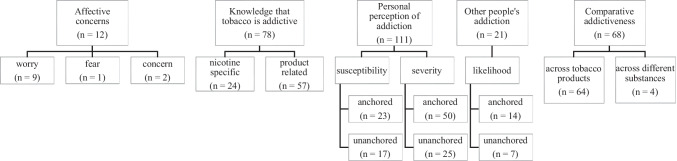
Thematic Groupings of Single Item Assessments

**Table 3:** Validated Measures Assessing Perceived Addictiveness

Article(s)	Prompt	Item(s)	Theme
Cano et al., 2018	In general, what do you think is the risk, if any, to smokers of experiencing the following because of smoking cigarettes…^1^	1. Being unable to quit cigarettes	Unanchored likelihood
		2. Feeling addicted to cigarettes	
		3. Having to smoke cigarettes to feel better	
		4. Feeling like they have to smoke cigarettes	
		5. Feeling like they can’t stop smoking cigarettes even though they know it is not good for them	
		6. Feeling unable to quit cigarettes	
Pokhrel et al., 2014; Pokhrel, Fagan, et al., 2018; Pokhrel, Lam, et al., 2018; Selekoğlu Ok et al., 2020	What is the likelihood that the following outcomes would happen to you if you used e-cigarettes? ^2^	1. Feel controlled by e-cigarettes	Anchored susceptibility
		2. Make it harder to quit e-cigarettes	
		3. Become addicted to e-cigarettes	

^1^ Perceived Risk Instrument; ^2^ E-cigarette Use Outcome Expectancies Scale

### Eligibility Criteria

Searches were conducted in May 2022, restricting results to peer-reviewed studies, published in English, focusing on human adults (18 +), and published in or after 1952 (the year the original DSM was published). Articles were included if they assessed perceptions of addiction related to nicotine and/or tobacco as much of the existent research generally prioritizes understanding how lay people appraise overall products, rather than just their addictive component (i.e., nicotine).

We excluded articles that were exclusively experimental (e.g., randomized controlled trials) that did not assess perceptions of nicotine addiction using survey methods. Next, we excluded qualitative studies that did not include a quantitative component assessing perceptions of nicotine addiction. Although qualitative research provides rich data, we were interested in whether there were any attempts of psychometrically evaluating perceptions of nicotine addiction that aligned within a perception of risk framework. We then excluded papers that used national tobacco survey data which utilize a single item to assess perceptions of nicotine addiction (e.g., Population Assessment of Tobacco and Health [PATH], National Adult Tobacco Survey [NATS]). Peer-reviewed publications using these data analyze the same cohort samples using the same respective single item assessment of perceptions of nicotine addiction. For this reason, we chose to only reference the overall dataset for PATH and NATS in Supplemental Material 1, instead of individual publications using these data (PATH = 21 papers; NATS = 3 papers). Fourth, we excluded papers examining only youth populations as some dimensions of addiction may apply differently among youth compared to adult populations [33], however assessments of perception of addiction within studies may have utilized youth-specific language among an adult-only sample. We also excluded papers that did not assess perceived addictiveness of tobacco, or its addictive component—nicotine – using survey methods. Lastly, we excluded papers that only utilized diagnostic measures of dependence (e.g., NDSS, etc.) as these are separate from *perceptions* of nicotine addiction.

### Papers Identified

The PubMed search returned 2,608 results; the PsycInfo search returned 4,876 results. Following the removal of duplicates, 5,859 papers remained. After reviewing titles and abstracts, and the addition of 10 articles from reference lists, 923 empirical studies remained that initially met our criteria.

## Results

Following full-text review, 671 papers were excluded because they met the exclusionary criteria discussed above, leaving 252 empirical articles that assessed perceptions of addiction using either single item measures or an existing, validated scale. We categorized single item assessments (*n* = 247) into reoccurring themes and aligned the items found in validated scales (*n* = 5) within a perception of risk framework (e.g., susceptibility, severity, etc.).

### Single Item Assessments

Some articles assessed perceptions of addiction with more than one question (but not a scale) and have been placed into multiple themes (Table 2). This practice was not very common, with 33 papers using two items, nine papers using three items, and two papers using four items to assess perceptions of addiction. There were no instances of a single-item question being placed into multiple themes. The definitions for each theme are described, and all examples of single item assessments can be found in Supplementary Material 1.

#### Affective Concern

Affective concerns were questions related to fear, worry, or concern over becoming addicted to tobacco products (*n* = 12).

##### Fear

Only one article assessed participants’ fear of becoming addicted to a tobacco product [34]. This is the only item that touches on fear of addiction, even across the validated measures.

##### Worry

 Nine articles assess a persons’ worry about becoming addicted to the nicotine found in various tobacco products [35–43]. Whereas the tobacco products may differ (e.g., waterpipe, cigarettes), the items specify the *nicotine* found in these products.

##### Concern

Only two papers assess a persons’ concern over becoming addicted to tobacco products [44, 45] (e.g., “Are you concerned about becoming addicted to [snus/smoking]”?).

#### Knowledge That Tobacco is Addictive

Items in this theme assess participant knowledge of the addictive properties found in tobacco products or acknowledgement that tobacco is addictive (e.g., “Nicotine is the addictive component of tobacco products”; *n* = 78). During item examination, the need to separate between nicotine knowledge and general product knowledge became evident. Not only is there variation in item construct in both subsections, but also response options, with some articles utilizing Likert scales and others dichotomizing their response options.

##### Nicotine Specific

Twenty-four of the 78 articles assessed the perceived addictiveness of nicotine [46–69]. One item (“Nicotine is addictive, regardless of whether ingested through e-cigarettes or regular cigarettes”) is part of a larger scale named Risks and Benefits of E-cigarettes (RABE) scale [46]. In some instances, articles investigated perceptions related tobacco products with lower nicotine concentration [57–59, 62]. Three of 4 articles were assessing the perceived addictiveness of a “low in nicotine” cigarette and utilize a five-point Likert scale; the remaining article used a descriptor of a hypothetical “clean” nicotine product and utilized a four-point Likert scale [62].

##### Product Related

Fifty-seven of the 78 articles focus on the general addictiveness of tobacco products [26, 47, 51, 59, 70–122]. There is a high level of heterogeneity in item structure with some articles assessing whether specific tobacco products (e.g., cigarettes, snus) are generally addictive or whether using specific products can lead to nicotine addiction. Furthermore, there are instances where items may be identical, but have differing response options such that one author assesses agreement [70] and another utilizes a true/false option [71].

#### Personal Perceptions of Addiction

Personal perceptions of addiction are related to one’s own assessment of their own addiction to tobacco products (e.g., “How addicted are you to [cigarettes/smoking]”?). This theme included a greater number of articles compared to other themes (*n* = 111). There is variation in item construct, with differences in whether the item is anchored to the respondents’ own behavior or general perceptions of the addictiveness of specific tobacco products. This was further categorized into perceived severity and susceptibility.

##### Anchored Susceptibility

Anchored susceptibility are questions related to self-perceived susceptibility (i.e., likelihood) of becoming addicted to tobacco if they [continue/were to] to smoke (*n* = 23) [35–43, 68, 123–135]. Tobacco products of interest include hookah/waterpipe, e-cigarettes, and combustible cigarettes. There is heterogeneity in item construct, with select articles specifying becoming addicted to the nicotine in tobacco products, whereas other articles refer to the overall product.

##### Unanchored Susceptibility

Unanchored susceptibility refers to general questions directed at the individual assessing their perceived susceptibility (i.e., likelihood) of becoming addicted to tobacco products (*n* = 17) [44, 136–150, 151]. Three of four articles examining waterpipe use separated perceived likelihood into 3 individual items: using hookah alone, socially, and occasionally [136–138]. One item was part of a larger scale (Perceived Health Risks scale) that assessed the perceived risk of developing various health outcomes due to JUUL use, general e-cigarette use, and cigarette use [44, 140–146] (only the question related to developing addiction is included in this review).

##### Anchored Severity

Anchored severity refers to self-perceived severity of addiction to tobacco (*n* = 50) [27, 37, 118, 130, 131, 152–196]. Most items are phrased to assess the extent to which a person believes they are addicted to [tobacco product(s)]. Only two papers assessed the extent to which a person was addicted specifically to nicotine [173, 181]. Response options to these items are highly varied ranging anywhere from 1–5, 1–100, and categorical options (*not at all* to *very addicted)*.

##### Unanchored Severity

Unanchored severity refers to general questions directed at the individual assessing how addictive various tobacco products are (*n* = 25) [25, 115, 191, 197–218]. There is relative homogeneity in item structure, with most differences being in available response options (e.g., three vs. seven-point Likert type scale, 0–100%, etc.). In each of these papers, the participants assessed the addictiveness of the tobacco product as a whole, not the nicotine content specifically.

#### Other People’s Addiction

Other people’s addiction are questions related to the extent to which another person could become or are addicted to tobacco products (e.g., “On the average, are most regular smokers addicted”; *n* = 21). There is heterogeneity in item construct with some articles asking about specific groups of people (e.g., Monks, youth), and others using general terms (i.e., “most people”). Articles assessing likelihood of youth of becoming addicted to tobacco were directed towards people aged 18 and older.

##### Anchored Likelihood

Anchored likelihood refers to general perceptions regarding the likelihood of others to become addicted to tobacco products after a given amount and/or time (e.g., half a pack a day; *n* = 14) [26, 111, 151, 195, 219–228]. Two articles focused on the likelihood of youth becoming addicted to tobacco [26, 111], but these questions were directed towards people aged 18 years and older. One article focused on a specific group of individuals (e.g., Monks) [228], and the remaining articles focused on a more general population. Item structure is highly varied, with main differences in how much tobacco must be consumed and the amount of time one must use a tobacco product before being considered “addicted”.

##### Unanchored Likelihood

Unanchored likelihood refers to perceived likelihood of others to become addicted to tobacco products (*n* = 7) [181, 185, 188, 229–232]. There is a high level of heterogeneity in item structure, with most authors asking a generalized question about others, and one author focusing on a particular ethnic group [231]. Only one of these articles was published within the past five years [230], suggesting that measurement of this construct may no longer be commonplace amongst researchers.

#### Comparative Addictiveness

Comparative addictiveness is defined as comparing the addictiveness of one tobacco product in relation to other products—including non-tobacco products (e.g., “E-cigarettes are less addictive than [combustible] cigarettes”; *n* = 68). Comparative substances ranged between other tobacco products (e.g., e-cigarettes, snus, hookah/waterpipe), nicotine replacement therapies (e.g., nicotine patches), and non-tobacco products (e.g., cocaine). Although the comparative substance varies across articles, the overall structure of the comparative question remains relatively similar. Significant differences in question structure were mostly regarding whether the two products are as addictive as one another, or if one was more addictive than the other.

##### Across Tobacco Products

Comparative addictiveness across tobacco products are direct comparisons of perceived addictiveness between two or more tobacco products (*n* = 64) [24, 38, 42–44, 57–59, 62, 74, 75, 93, 96, 122, 126, 139, 150, 170, 171, 190, 210, 214, 233–274]. Most, if not all, articles use cigarettes as the referent category, with select papers comparing one type of cigarette to another (e.g., menthol vs. non-menthol) [250, 254, 266]. A handful of articles compare smoking cigarettes to nicotine replacement therapies (e.g., nicotine gum, nicotine patches, etc.) [57–59, 267, 268]. A majority of the articles compare the addictiveness of e-cigarette products to cigarettes, suggesting the growing need to understand perceptions of nicotine addiction of newer tobacco products.

##### Across Different Substances

Comparative addictiveness across different substances are direct comparisons of perceived addictiveness between a tobacco product and another substance (e.g., heroin; *n* = 4) [184, 226, 275, 276]. Given the limited number of articles that assess perceptions of nicotine addiction in this way, one might expect some level of uniformity between the phrasing of the items, or at the very least, the same comparative substance. However, the heterogeneity in item structure highlights the discrepancy in how individual investigators assess perceptions of addiction and how this contributes to non-standardized assessments of important constructs.

### Validated Scales Assessing Perceptions of Nicotine Addiction

Only five articles assess perceptions of nicotine addiction using a validated measure. Cano et al. [277] utilize the Perceived Risk Instrument which includes an addiction subscale. Respondents are asked to reflect on the risk of smokers’ experiencing six distinct experiences related to cigarette use (e.g., “feeling unable to quit cigarettes”; see Table 3). These items are an appraisal of other people’s behaviors/feelings and fit within the theme of unanchored likelihood. Response options followed a Likert type format from 1 = *no risk* to 5 = *very high risk* and had high internal reliability within their sample (Cronbach’s alpha = 0.98)*.*

Four articles [278–281] utilize the E-cigarette Use Outcomes Expectancies Scale (EUOES) which contains an addiction subscale comprised of three items. These items assess an individuals’ personal susceptibility of becoming addicted to e-cigarettes, including how difficult it would be to quit using e-cigarettes, and how controlled they would feel by their e-cigarette devices if they were to use them. Response options ranged from 1 = *completely unlikely* to 10 = *completely likely.* Within validated samples, the EUOES addiction subscale held sufficient internal reliability between 0.838 to 0.87.

## Discussion

This scoping review provides a comprehensive summary of existing empirical measurement of perceptions of nicotine addiction. Full text analysis demonstrates extreme deviation in single item assessments and validated measures assessing perceptions of nicotine addiction. Moreover, it highlights the discrepancies in individuals’ *perceptions* regarding nicotine addiction to diagnostic assessments of addiction severity/level of dependence.

The heterogeneity in two validated measures of perceptions of nicotine addiction (e.g., Perceived Risk Instrument, EUOES) is reflective of the complexity of addiction and how people perceive the way in which nicotine addiction manifests, despite the fact that we have a well-established criteria to diagnose nicotine dependence/addiction severity. Diagnostic assessments are designed to encapsulate the breadth of experiences when addicted; in contrast, psychological researchers emphasize the intensity of some of these experiences. It is essential to marry these ideas together to fully reflect the experiences of one who is addicted. Specifically, by measuring perceptions of nicotine addiction in line with diagnostic dimensions, psychological research can determine how the perceptions of addiction dimensions contribute to health decision making—both *which* dimensions contribute and *how much* they contribute.

Of particular interest is the lack of items assessing perceptions related to withdrawal and tolerance. The experience of tobacco withdrawal during cessation attempts are well documented [282], and it could be argued that one reason many tobacco users fail initial cessation attempts are, in part, due to the severity of their withdrawal. For example, difficulty sleeping when abstaining from tobacco use may not be perceived as an indicator of withdrawal, despite this being established in the DSM-5 [12]. Moreover, perceived experiences of withdrawal could be conflated with craving, further impacting the layperson’s comprehension of nicotine addiction. It is therefore imperative to assess perceptions related to withdrawal as there is a growing body of literature to suggest non-tobacco users may underestimate the difficulty of quitting once they begin using tobacco [283, 284]. Measurement of perceptions of nicotine addiction related to tolerance are also warranted, particularly as the customizability of nicotine concentration levels becomes more mainstream. Although users may not be aware of the exact nicotine content in their tobacco products [285], they are more likely to be aware of the frequency in which they use the product (e.g., how many cigarettes they smoke per day, or how much e-liquid they use). Whereas users may not be directly aware of their increasing nicotine use, questions can be developed to determine whether they are seeking nicotine to satiate their needs as their tolerance builds up.

### Current Understanding of Perceptions of Nicotine Addiction

Existent work is primarily focused on perceived severity and susceptibility to addiction, with most research assessing whether a certain tobacco product is addictive. However, there is a dearth of literature describing the construct of addiction and how it is perceived by individuals. In this regard, there is a disconnect between the literature focused on understanding the addictiveness of nicotine and what nicotine addiction means to the lay person. For example, single items may assess whether a tobacco product is addictive; conversely, scales may assess an individual’s risk of becoming addicted to those products. However, these items and scales do not meaningfully describe what addiction looks like for the lay person. To understand how people appraise addiction, further investigation is needed to disentangle its physical, psychological, and social components. Specifically, when considering nicotine addiction, do people focus primarily on withdrawal symptoms, craving, or inability to quit despite social and economic costs? Understanding lay people’s comprehension of nicotine addiction and how they view nicotine addiction is instrumental in developing targeted interventions for both prevention and intervention-based research.

### Strengths and Limitations

To our knowledge, this is the first comprehensive attempt at summarizing measures assessing perceptions of nicotine addiction using a perceived risk framework. Secondly, our organization of items clearly highlight the divergence in construct measurement between independent researchers and discusses how this can create issues in applied settings. Finally, our review spans research over seven decades, which is helpful to understand how the tobacco landscape has shifted. Since 2013, we have seen an increase in research examining alternative tobacco products (e.g., e-cigarettes). Our review found that research typically assesses people’s perceptions of these newer products in comparison to more well-known products (i.e., cigarettes), despite the addictive component in both tobacco products being nicotine.

One limitation of this review is the exclusion of items used with youth populations. However, adolescence is a developmental period marked with relatively high rates of substance use and substance use disorder. Some clinical criteria such as tolerance and withdrawal apply differently among youth, therefore the need to separate these groups is warranted [33]. Additionally, our search string did not specify electronic cigarettes as a substance, which may have inadvertently excluded articles that investigate e-cigarette specific related perceptions of addiction. However, articles referenced in Supplemental Material 1 suggest that existing work on e-cigarette related perceptions are usually in comparison to the perceived addictiveness of combustible cigarettes. Finally, we did not investigate whether there were thematic differences in participants’ tobacco use status within included articles, however future research can build upon on our review and conduct a more granular review to determine whether these differences exist.

### Future Directions/Implications

Although scales assessing perceived addictiveness of nicotine do exist, there is room for improvement. The Perceived Risk Instrument has yet to be tested outside of a predominantly non-Hispanic White sample [277]. It should be noted, the authors were either employed or contracted through Philip Morris International, one of the largest tobacco companies, which may help explain the lack of retest reliability in more diverse populations. Similarly, the EUOES remains to be tested outside of university student samples. It is possible that people may share similar perceptions of addiction to nicotine, however, the weight of these perceptions may differ across groups (i.e., college students vs. non-college students). This scoping review is meant to serve as a starting point of creating a standardized measure designed to assess perceptions of nicotine addiction that are inclusive of both psychological constructs (e.g., susceptibility, severity, likelihood, etc.) as well as diagnostic assessments of addiction (e.g., craving, withdrawal, tolerance, etc.).

The overwhelming number and diversity of single item measures assessing this complex construct raise concerns about how we can draw conclusions across different studies using non-standardized measures. As demonstrated, there is also a concern about what facets of addiction are more strongly represented in the research and unfairly weight our understanding of addiction as a whole. As of now, it is unclear if conflicting research findings in the literature are truly conflicting or if these single-item measures are assessing different facets of addiction. The findings from this review may inform emerging scientists interested in examining specialized domains of tobacco regulatory science (e.g., addiction) by encouraging standardization of items assessing perceptions of nicotine addiction in psychological research [286]. In this regard, aligning perceptions of nicotine addiction with defined risk perception dimensions may lend support for tailored prevention and cessation interventions. Moreover, understanding *which* dimensions of addiction and *how much* they contribute to people’s understanding of nicotine addiction.

## Conclusions

This review displays the lack of standardization among measures assessing perceptions of nicotine addiction, despite claiming to assess the same construct, which contributes to the lack of understanding of nicotine addiction among lay people. Single item assessments of perceptions of nicotine addiction are highly varied, and validated measures assess different dimensions of perceived risk (e.g., severity vs susceptibility). To better understand the impact of perceived addictiveness on health behavior decision making, it is critical to develop a more comprehensive measure that aligns psychological research and diagnostic assessment.

## Key References

- Copeland, A. L., Peltier, M. R., & Waldo, K. (2017). Perceived risk and benefits of e-cigarette use among college students. Addictive Behaviors, 71, 31–37. https://doi.org/10.1016/j.addbeh.2017.02.005.
  - A well-cited scale that includes one question assessing the perceived addictiveness of nicotine via e-cigarettes versus cigarettes.
- Hatsukami, D. K., Vogel, R. I., Severson, H. H., Jensen, J. A., & O’Connor, R. J. (2016). Perceived health risks of snus and medicinal nicotine products. Nicotine & Tobacco Research: Official Journal of the Society for Research on Nicotine and Tobacco, 18(5), 794–800. https://doi.org/10.1093/ntr/ntv200.
  - A well-cited scale that includes one question assessing the perceived risk of developing addiction due to JUUL use.

## Supplementary Information

Below is the link to the electronic supplementary material.

Supplementary file1 (DOCX 294 KB)

Supplementary file2 (DOCX 97 KB)

Supplementary file3 (DOCX 26.4 KB)

## Data Availability

No datasets were generated or analysed during the current study. All remaining references included in this review can be found in Supplemental Material 3.
