# Supplementary material for: Cravings, Control, and Cessation: A Scoping Review of Perceptions of Nicotine Addiction
Source: Curr Addict Rep. 2025 Jul 11;12(1):66. doi: 10.1007/s40429-025-00673-4 (PMC12254085; doi:10.1007/s40429-025-00673-4)
Supplement: Supplementary file 1 — Supplementary file1 (DOCX 294 KB) [file 40429_2025_673_MOESM1_ESM.docx]

**Supplemental Material 1**. Single item assessments of perceived addictiveness of nicotine (products)

| **Citation** | **Item(s)** | **Tobacco Product** |
| --- | --- | --- |
| ***Affective Concerns* (n = 12)** | | |
| **Worry (n = 9)** | | |
| (Lipkus et al., 2011, 2014, 2015, 2017; Lipkus & Mays, 2018; Lipkus & Noonan, 2017; Lipkus & Sanders, 2021; Mays, Tercyak, et al., 2016; Phan et al., 2021) | - How worried are you about becoming addicted to nicotine in waterpipe if you continue to smoke? - How worried are you about becoming addicted to nicotine in cigarettes if you continue to smoke? - How worried would you be about becoming addicted to nicotine in waterpipe in your lifetime if you were to smoke it and not quit? | Waterpipe (i.e., hookah, narghile, shisha) |
| **Fear (n = 1)** | | |
| (Etter & Bullen, 2011) | - Afraid of becoming addicted to e-cigarette? | E-cigarette |
| **Concern (n = 2)** | | |
| (Hatsukami et al., 2016) | - Are you concerned about becoming addicted to snus? - Are you concerned about becoming addicted to nicotine gum? | Snus  Nicotine gum |
| (Lim et al., 2019) | - Are you concerned about becoming addicted to smoking? | N/A |
| ***Knowledge that Tobacco is Addictive* (n = 78)** | | |
| **Nicotine Specific (n = 24)** | | |
| (Copeland et al., 2017; Garey, Smit, et al., 2019; Mayorga et al., 2019, 2020; Roys et al., 2020; Smit et al., 2022; Zvolensky et al., 2019, 2019; Zvolensky, Mayorga, et al., 2019a, 2019b; Zvolensky, D’Souza, et al., 2020) | - Nicotine is addictive, regardless of whether ingested through e-cigarettes or regular cigarettes | Nicotine |
| (Cummings, Hyland, Giovino, et al., 2004; Villanti, Naud, West, Pearson, Wackowski, Hair, et al., 2019; Villanti, Naud, West, Pearson, Wackowski, Niaura, et al., 2019) | - The claim that a cigarette brand is low in nicotine means that it is less addictive | Nicotine |
| (Danishevski et al., 2008) | - Is nicotine addictive? | Nicotine |
| (Lynam et al., 2012) | - If I use nicotine replacement therapy, I will still be addicted to nicotine | Nicotine |
| (Morphett et al., 2021) | - Using clean nicotine products long-term is bad because it maintains addiction | Nicotine |
| (Morrell et al., 2008) | - The nicotine in cigarettes is addictive | Nicotine |
| (Pacek et al., 2017) | - Nicotine is the addictive component of tobacco products | Nicotine |
| (Patel et al., 2019) | - Nicotine exposure from e-cigarettes (including JUUL) during adolescence can cause addiction | Nicotine |
| (Reddy et al., 1996) | - Nicotine and condensate in tobacco products cause addiction | Nicotine |
| (Saddichha et al., 2010) | - Nicotine is the most addictive drug | Nicotine |
| (Yang, Owusu, et al., 2019a; Yang & Popova, 2020) | - Nicotine is the main substance in tobacco that makes people become addicted to tobacco products | Nicotine |
| **Product Related (n = 57)** | | |
| (Abdel-Qader & Al Meslamani, 2021; Mark et al., 2015) | - E-cigarette [smoking] can be addictive | E-cigarette |
| (Agaku & Filippidis, 2014) | - Is tobacco use addictive? | Tobacco |
| (Aghar et al., 2020) | - E-cigarettes are not addictive | E-cigarette |
| (Amin et al., 2010) | - Ssisha smoking is easier to quit and causing no addiction | Waterpipe |
| (Anjum et al., 2008) | - Opinion whether water pipe is addictive | Waterpipe |
| (Aryal & Lohani, 2011; Murphy-Hoefer et al., 2004; Seigers & Terry, 2011; Tercyak et al., 2005) | - Can people get addicted to using tobacco just like they can get addicted to using cocaine or heroin? | Tobacco |
| (Budd & Preston, 2005; Ceballos et al., 2009; Minhas & Rahman, 2009; Netemeyer et al., 2005; Siqués et al., 2006; West & Hargreaves, 1995) | - Smoking is addictive | N/A |
| (Azab et al., 2013; Brownson et al., 1992; Cummings et al., 1991; Harrell, Marquinez, et al., 2015) | - Cigarettes are addictive | Cigarettes |
| (Garey, Mayorga, et al., 2019; Garey, Smit, et al., 2019; Harrell, Marquinez, et al., 2015; Harrell, Simmons, et al., 2015; Mayorga et al., 2021; Mays et al., 2019; Smit et al., 2022; Zvolensky, Shepherd, et al., 2020) | - E-cigarettes is/were addictive | E-cigarettes |
| (N. H. Gottlieb et al., 1992) | - Smokeless tobacco is addictive | Smokeless tobacco |
| (Kaufman et al., 2014) | - Snus is addictive | Snus |
| (Harrell, Marquinez, et al., 2015) | - Nicotine replacement therapy is addictive | Nicotine replacement therapy |
| (Baig et al., 2016) | - Smoking is an addiction | N/A |
| (Berry & Burton, 2019) | - Based on the information contained in the warning, e-cigarettes are addictive | E-cigarettes |
| (Berry et al., 2017b, 2017a) | - Electronic cigarettes are addictive - Traditional cigarettes are addictive | Electronic and traditional cigarettes |
| (Braun et al., 2012; Dani et al., 2015; Fevrier et al., 2018) | - Hookah is addictive | Waterpipe |
| (Chaaya, Jabbour, et al., 2004; Chaaya, Roueiheb, et al., 2004) | - Cigarettes contain addictive substances - Argileh contains addictive substances | Waterpipe  Cigarettes |
| (Chassin et al., 2010) | - Smoking is an addictive behavior | N/A |
| (Cummins et al., 2016) | - E-cigarettes are highly addictive | E-cigarettes |
| (Galimov et al., 2021) | - In your opinion, do e-cigarettes and other vaping products contribute to young people becoming addicted to nicotine? | Electronic tobacco products |
| (Glasser et al., 2020; Rath et al., 2018) | - Menthol in cigarettes is linked to becoming a regular smoker | Menthol cigarettes |
| (J. C. Gottlieb et al., 2013) | - Chewing tobacco leads to nicotine addiction | Chewing tobacco |
| (Hakim et al., 2018) | - Belief that cigarettes are addictive | Cigarettes |
| (Janik-Koncewicz et al., 2012) | - Tobacco smoking can lead to psychological addiction as strong as drug addiction | Tobacco |
| (Lydon et al., 2016) | - How likely is it that smoking causes cigarette addiction? - How likely is it that cigarette addiction causes smoking? | Cigarette |
| (Majeed et al., 2018) | - Can people become addicted to little cigars and cigarillos? | Little cigars  Cigarillos |
| (Pillitteri et al., 2020) | - Is Camel snus, which contains nicotine, addictive? | Snus |
| (Sarfraz et al., 2018) | - Do you think e-cigarettes are addictive? | E-cigarettes |
| (Scott et al., 2015) | - Smoking leads people to become addicted to cigarettes for life | N/A |
| (Seng et al., 2020) | - In your opinion, does smoking/tobacco use cause addiction to other drugs? | Tobacco |
| (Stearns et al., 2012) | - Smoking is physically addictive | N/A |
| (Sterling et al., 2017) | - Are little cigars and cigarillos addictive? | Little cigars  Cigarillos |
| (Villanti, Naud, West, Pearson, Wackowski, Niaura, et al., 2019) | - It is easy to get addicted to nicotine gum - It is easy to get addicted to e-cigarettes - The claim that an e-cigarette brand is low in nicotine means that it is less addictive | Nicotine gum  E-cigarettes |
| (Wainwright et al., 2019) | - Smoking leads to nicotine addiction | N/A |
| (Zgliczyński et al., 2019) | - You can become addicted to the e-cigarette | E-cigarette |
| ***Personal Perception of Addiction* (n = 111)** | | |
| **Anchored Susceptibility (n = 23)** | | |
| (Barker et al., 2019; Tan et al., 2016) | - If I were to use an e-cigarette or other vaping device every day, I would get addicted | E-cigarette |
| (Differding et al., 2022) | - Indicate what you believe your risk would be for addiction if you regularly smoked/used cigarettes with 95% less nicotine | Cigarette |
| (Dixit et al., 2021) | - If I vape or use e-cigarettes every day, I will become addicted | E-cigarette |
| (Hawash et al., 2022) | - Perception on addiction to waterpipe smoking (yes addictive, not addictive, maybe addictive) | Waterpipe |
| (Koski-Jännes et al., 2012) | - How great the risk of developing dependence (aka becoming addicted) when trying smoking? | N/A |
| (Lipkus et al., 2011, 2014; Mays, Tercyak, et al., 2016; Roberts & Ferketich, 2020)  (Lipkus & Mays, 2018)  (Lipkus et al., 2015, 2017; Lipkus & Noonan, 2017; Lipkus & Sanders, 2021; Roberts & Ferketich, 2020)  (Phan et al., 2021) | - What do you think is the chance of you becoming addicted to nicotine in tobacco from waterpipe if you continue to smoke? - What do you think is the chance of you becoming addicted to nicotine in tobacco from waterpipe if you did not quit? - What do you think is your chance of becoming addicted to nicotine in tobacco from waterpipe if you were to smoke it? - What do you think is your chance of becoming addicted to nicotine in tobacco from waterpipe if you were to smoke it and not quit? | Waterpipe |
| (Moss & Bould, 2009) | - I do smoke, but am not addicted to smoking | N/A |
| (Pechacek et al., 2018; Popova et al., 2018; Yang, Owusu, et al., 2019b; Yang, Spears, et al., 2019; Yang & Popova, 2020) | - Imagine you just began smoking cigarettes everyday, what do you think are your chances of becoming addicted? - Imagine you just began smoking cigarettes once in a while, what do you think are your chances of becoming addicted? - Imagine you just began smoking cigarettes on occasion, what do you think are your chances of becoming addicted? | Cigarettes |
| (Presson et al., 2002) | - If I smoke, I will be hooked | N/A |
| **Unanchored Susceptibility (n = 17)** | | |
| (Abughosh et al., 2012; Azab et al., 2010; S. Y. Smith-Simone et al., 2008) | - What is the likelihood of getting addicted to waterpipe when using the product alone? - What is the likelihood of getting addicted to waterpipe when using the product socially? - What is the likelihood of getting addicted to waterpipe when using the product occasionally? | Waterpipe |
| (Brożek et al., 2017) | - Possibility of becoming addicted to e-cigarettes | E-cigarette |
| (DeAtley et al., 2020; Denlinger-Apte et al., 2017; Hatsukami et al., 2013, 2016; O’Connor et al., 2016; Pacek et al., 2019, 2021; Wolfson et al., 2014) | - On a scale from 1 to 10, rate your perceived risk of developing addiction as a result of your (a) JUUL use, (b) general ENDS use, and (c) cigarette smoking | JUUL  Electronic Nicotine Delivery Systems  Cigarettes |
| (Hoetger et al., 2019) | - What do you think is the likelihood of addiction is when using this product? | E-cigarette |
| (Landry et al., 2019) | - Do you believe you are addicted to or dependent upon vaping? | Electronic Nicotine Delivery Systems |
| (Lopes-Costa & Amato-Vealey, 2016) | - Do you believe you are addicted to smoking? | N/A |
| (Majek et al., 2021) | - Do you think you can become addicted to Heated Tobacco Products? | Heated tobacco products |
| (Waters et al., 2016) | - If I have not yet become addicted to nicotine then I never will | Nicotine |
| **Anchored Severity (n = 50)** | | |
| (Alam et al., 2020) | - In your opinion, how hooked are you on smoking waterpipe? | Waterpipe |
| (Balmford & Borland, 2008; Hughes & Naud, 2016b) | - I am too addicted to be able to quit | Tobacco |
| (M. B. Berg et al., 2017; Chaiton et al., 2017; Martin, 1990; Sabogal et al., 1989; Yunus & Khan, 1997) | - How addicted are you to smoking? | N/A |
| (Ashley et al., 2001; T. V. Cooper et al., 2010; Edwards et al., 2010; Hughes & Naud, 2016a; Lenk et al., 2009; Li et al., 1988; Lyna et al., 2002; Marin et al., 1990; Sendzik et al., 2011; Torchalla et al., 2011) | - How addicted are you to cigarettes? | Cigarettes |
| (Etter, 2015, 2016) | - How addicted are you to e-cigarettes? | E-cigarettes |
| (Maziak et al., 2004) | - How hooked are you on narghile? | Waterpipe |
| (Schippers & Cox, 1994) | - How addicted are you to nicotine? | Nicotine |
| (Seng et al., 2020) | - How addicted are you to tobacco? | Tobacco |
| (Yates et al., 2014) | - How addicted are you to cigarillos? | Cigarillos |
| (Camara-Medeiros et al., 2021) | - Would you say that you are ‘very addicted to vaping,’ ‘somewhat addicted to vaping,’ ‘not at all addicted to vaping,’ or ‘I don’t know’? | Electronic Nicotine Delivery Systems |
| (Chen, 2009) | - If you are not allowed to smoke, you will feel [unaffected – very uncomfortable] | N/A |
| (DiFranza et al., 2012) | - Have you ever felt like you were addicted to tobacco? | Tobacco |
| (Okoli et al., 2016) | - Have you ever felt like you were addicted to smoking? | N/A |
| (Eiser et al., 1985; Eiser & Van der Pligt, 1986) | - How addicted do you think you are to smoking? | N/A |
| (Farrimond, 2017) | - I dislike the fact that I’m still addicted to nicotine | Nicotine |
| (Gillies et al., 1989) | - What is the primary reason you smoke? [Addiction] | N/A |
| (Gilpin et al., 2002; Palinkas et al., 1993) | - I believe that I am addicted to cigarettes | Cigarettes |
| (Levinson et al., 2007; Perski et al., 2019; Vander Martin et al., 1990; Zinser et al., 2011) | - I am addicted to cigarettes | Cigarettes |
| (Miller et al., 2014) | - I am addicted to cigarettes - I am addicted to smokeless tobacco | Cigarettes  Smokeless tobacco |
| (Perski et al., 2019) | - I am addicted to smoking | Smoking |
| (Stippekohl et al., 2012) | - Do you think you are addicted to cigarettes? | E-cigarettes  Cigarettes |
| (Goniewicz et al., 2013) | - Do you think you are addicted to e-cigarettes? | E-cigarettes |
| (Thawal et al., 2022) | - Do you think you might be addicted to tobacco? | Smokeless tobacco  Cigarettes, bidis, etc. |
| (Konkolÿ Thege, Colman, El-guebaly, et al., 2015) | - Thinking back over your life, have you ever personally had a problem with tobacco? | Tobacco |
| (Lipkus et al., 2015) | - To what extent do you think you are addicted to cigarettes? | Cigarettes |
| (Moss & Bould, 2009) | - For me, smoking a cigarette is like a drug addiction | Cigarettes |
| (United States Department of Health and Human Services et al., 2021) | - Do you consider yourself to be addicted to [tobacco/products]? | Tobacco |
| (Mostafa, 2020) | - Do you consider yourself addicted to waterpipe tobacco smoking? | Waterpipe |
| (Pechacek et al., 2018; Weinstein et al., 2004) | - Do you consider yourself addicted to cigarettes? | Cigarettes |
| (Vu et al., 2019) | - Do you believe you are addicted to or dependent upon tobacco use? | Tobacco |
| (Weinstein et al., 2004) | - Compared to the average smoker, would you say that you are more addicted to cigarettes, less addicted to cigarettes, or about the same? | Cigarettes |
| **Unanchored Severity (n = 25)** | | |
| (Alizaga et al., 2020; C. J. Berg et al., 2015, 2018, 2021; Jiang et al., 2019; Perks et al., 2019; Pillitteri et al., 2020) | - How addictive do you think the following tobacco products are? | Cigarettes  Little cigars and cigarillos  Smokeless tobacco  E-cigarettes  Waterpipe  Any tobacco |
| (Blomqvist, 2012; Calhoun, 1974) | - How addictive is tobacco? | Tobacco |
| (Case et al., 2020; M. Cooper et al., 2017, 2018; North et al., 2021) | - How addictive are [ENDS products/JUUL/pod vapes]? | Electronic nicotine products |
| (Creamer et al., 2016) | - How addictive is hookah? | Waterpipe |
| (Garbutt et al., 2015; Jongenelis et al., 2019; Kimber et al., 2018, 2020; Kolar et al., 2014; Mays, Smith, et al., 2016; Vogel et al., 2021; Webb Hooper & Kolar, 2017) | - How addictive are cigarettes? | Cigarettes |
|  | - How addictive are e-cigarettes? - How addictive do you think e-cigarettes are? | E-cigarettes |
| (Havermans et al., 2021) | - To what extent do you think the following tobacco products are addictive? | Cigarillos  Heated tobacco products  Nicotine pouches |
| (Center for Disease Control and Prevention, 2015) | - Overall, would you say [tobacco product] smoking/use is? | Cigarettes  Cigars  Smokeless tobacco |
| (Thawal et al., 2022) | - Do you think the following tobacco products are addictive? | Cigarettes  Smokeless tobacco |
| ***Other People’s Addiction* (n = 21)** | | |
| **Anchored Likelihood (n = 14)** | | |
| (Arnett, 2000) | - Most people who smoke for a few years become addicted and can’t stop | Cigarettes |
| (Ashley et al., 2000) | - Daily smokers are addicted | N/A |
| (Chassin et al., 2007; Konkolÿ Thege, Colman, el-Guebaly, et al., 2015) | - How addictive do you believe smoking cigarettes are for “most people”? | Cigarettes |
| (Cunningham, 2012) | - There is a high risk of becoming addicted if someone tried cigarettes? | Cigarettes |
| (Eiser et al., 1977) | - On average, do you think that smokers are addicted to cigarettes? | Cigarettes |
| (Glasser et al., 2020; Rath et al., 2018) | - Youth smoking menthol cigarettes are more likely to become addicted to smoking | Cigarettes |
| (Sadava & Weithe, 1985) | - On average, are most regular smokers addicted? | Cigarettes |
| (Smith et al., 2011) | - A person can become addicted to cigarettes within weeks of starting to smoke | Cigarettes |
| (Vigna-Taglianti et al., 2019) | - One needs to smoke several cigarettes a day to become addicted | Cigarettes |
| (Waters et al., 2016) | - If signs of nicotine addiction haven't appeared by the time someone smokes a TOTAL of 5 packs of cigarettes, they will never become addicted - People can become addicted to cigarettes even if they only smoke one or two cigarettes a day - People can become addicted to cigarettes even if they only smoke one or two cigarettes a week - People can become addicted to cigarettes even if they only smoke occasionally with friends | Cigarettes |
| (Weinstein et al., 2004) | - If a teenager starts smoking half a pack of cigarettes a day, how long do you think it takes for them to show signs of nicotine addiction? | Cigarettes |
| (Yel et al., 2013) | - Monks should not accept cigarettes as an offering; it is an addictive offering that gives no benefit to the offerer | Cigarettes |
| **Unanchored Likelihood (n = 7)** | | |
| (Farrimond, 2017) | - Vapers and smokers have addictive personalities | N/A |
| (Levinson et al., 2007) | - People who smoke cigarettes [regularly] are addicted | Cigarettes |
| (Zinser et al., 2011) | - People who smoke cigarettes regularly are addicted to nicotine | Cigarettes |
| (Majeed et al., 2017) | - Do you think people can become addicted to [e-cigarettes/electronic vapor products]? | E-cigarettes  Electronic vapor products |
| (Meijer & Chavannes, 2021) | - Smokers continue to smoke because they are addicted to tobacco | tobacco |
| (Rahman et al., 2015) | - In your opinion, why do Bangladeshi people use smokeless tobacco products? | Smokeless tobacco |
| (Wackowski et al., 2019) | - How likely is someone to become addicted to e-cigarettes? | E-cigarettes |
| ***Comparative Addictiveness* (n = 68)** | | |
| **Across Tobacco Products (n = 64)** | | |
| (Al-Naggar et al., 2014; Al-Naggar & Saghir, 2011) | - Addiction to shisha as compared to cigarettes | Cigarettes vs. waterpipe |
| (Amin et al., 2010) | - If I have to smoke, I would use shisha because it is less harmful and less addictive compared to cigarettes | Cigarettes vs. waterpipe |
| (Anjum et al., 2008; Asfar et al., 2005) | - Belief about the addictive effects of waterpipe compared to cigarettes | Cigarettes vs. waterpipe |
| (Aqeeli et al., 2022; Choi et al., 2018; Choi & Forster, 2013b, 2014; Correa et al., 2018; Hershberger et al., 2017; Lee et al., 2018; Melin et al., 2018; Nicksic et al., 2017; Peters et al., 2015; Pokhrel et al., 2016; Stein et al., 2015; Trumbo, 2018; Zgliczyński et al., 2019) | - E-cigarettes are less addictive than cigarettes | Cigarettes vs. e-cigarettes |
| (Borgan et al., 2013; Ward et al., 2005) | - Waterpipe is more addictive than cigarettes | Waterpipe vs. cigarettes |
| (Brennan et al., 2015) | - How addictive are menthol cigarettes compared to nonmenthol cigarettes? | Menthol cigarettes vs. non-menthol cigarettes |
| (Brożek et al., 2017) | - Compared to a regular cigarette, how addictive are e-cigarettes? | Cigarettes vs. e-cigarettes |
| (Byron et al., 2019; Morgan & Cappella, 2021) | - Compared to current cigarettes, how addictive do you think the [tobacco product] would be? | E-cigarettes vs. snus vs. heated tobacco products vs. cigarettes |
| (Choi & Forster, 2013a) | - Snus is less addictive than cigarettes | Cigarettes vs. snus |
| (Cummings, Hyland, Bansal, et al., 2004) | - Are light cigarettes more likely, about the same, or less likely to cause someone to become addicted as regular cigarettes? | Light cigarettes vs. regular cigarettes |
| (Cummings, Hyland, Giovino, et al., 2004; Villanti, Naud, West, Pearson, Wackowski, Hair, et al., 2019; Villanti, Naud, West, Pearson, Wackowski, Niaura, et al., 2019) | - Are nicotine patches more likely, about the same, or less likely to cause someone to become addicted compared to regular cigarettes? - Is nicotine gum more likely, about the same, or less likely to cause someone to become addicted compared to regular cigarettes? | Cigarettes vs. nicotine replacement therapy |
| (Dixit et al., 2021; Rass et al., 2015) | - Are e-cigarettes more, equally, or less addictive than regular cigarettes? | Cigarettes vs. e-cigarettes |
| (Egnot et al., 2017) | - I believe the addictive potential of an electronic cigarette compared to traditional cigarettes is | Cigarettes vs. e-cigarettes |
| (Etter, 2015, 2016) | - Addiction to e-cigarette compared to former addiction to tobacco | Cigarettes vs. e-cigarettes |
| (Goniewicz et al., 2013; Rahman et al., 2022) | - Do you think that e-cigarettes are addictive? | Cigarettes vs. e-cigarettes |
| (Hatsukami et al., 2016) | - [Do you consider] the study product higher or lower risk [for addiction] than cigarettes? | Cigarettes vs. nicotine gum  Cigarettes vs. snus |
| (Hefner et al., 2019) | - Do you think electronic cigarettes are as addictive as tobacco cigarettes? | Cigarettes vs. e-cigarettes |
| (Heinz et al., 2013) | - Compared to smoking cigarettes, how likely is smoking hookah to cause addiction? | Cigarettes vs. waterpipe |
| (Hughes et al., 2005; Shadel et al., 2006) | - [Eclipse/Quest] is less addictive than cigarettes | Branded cigarettes vs. regular cigarettes |
| (Jongenelis et al., 2019) | - Which of the following statements best describes how addictive you consider e-cigarettes compared to tobacco cigarettes...” (less addictive, equally addictive, more addictive, don't know) | Cigarettes vs. e-cigarettes |
| (Kaufman et al., 2014) | - Do you believe that snus is less or more addictive than ordinary cigarettes? | Cigarettes vs. snus |
| (Lipkus et al., 2017; Mays, Tercyak, et al., 2016; Noonan & Patrick, 2013; Phan et al., 2021; S. Smith-Simone et al., 2008) | - Compared to regular cigarettes, how addictive do you think waterpipe tobacco use is? | Cigarettes vs. waterpipe |
| (Lund & Scheffels, 2014) | - Compare the health risks…of developing nicotine addiction from snus use and smoking | Smokable tobacco vs. snus |
| (Majek et al., 2021) | - Compared to regular cigarettes, how addictive are heated tobacco products? | Cigarettes vs. heated tobacco products |
| (Mays, Moran, et al., 2016) | - How addictive are cigarettes compared to snus? | Cigarettes vs. snus |
| (Mays et al., 2019; Mays, Smith, et al., 2016) | - Do you believe that e-cigarettes are less or more addictive than ordinary cigarettes? - How addictive are cigarettes to a person’s health relative to e-cigarettes? | Cigarettes vs. e-cigarettes |
| (Mercincavage et al., 2019) | - Cigarettes that are lower in nicotine are less addictive than regular cigarettes | Low nicotine cigarettes vs. regular cigarettes |
| (Morphett et al., 2021) | - There is no point switching from smoking cigarettes to using clean nicotine products long-term because this is just swapping one addiction for another | Cigarettes vs. clean nicotine products |
| (Moysidou et al., 2016) | - How addictive is nicotine replacement therapy compared to smoking? | Nicotine replacement therapy vs. cigarettes |
| (Perman-Howe et al., 2022; Wilson et al., 2019) | - Do you think e-cigarettes/vaping devices (with nicotine) are more addictive than tobacco cigarettes, less addictive, or are they equally addictive? - Do you think heat-not-burn products are more addictive than tobacco cigarettes, less addictive, or are they equally addictive? - Do you think nicotine replacement therapies are more addictive than tobacco cigarettes, less addictive, or are they equally addictive? - Do you think e-cigarettes/vaping devices (with nicotine) are more addictive than nicotine replacement therapies such as gums or patches, less addictive, or are they equally addictive? | Cigarettes vs. e-cigarettes  Cigarettes vs. heated tobacco products  Cigarettes vs. nicotine replacement therapies  E-cigarettes vs. nicotine replacement therapies |
| (Primack et al., 2008) | - Would you say that smoking from a waterpipe is more addictive or less addictive than smoking regular cigarettes? | Cigarettes vs. waterpipe |
| (Queloz & Etter, 2021) | - How addictive are tobacco vaporizers compared to combustible cigarettes? | Cigarettes vs. tobacco vaporizers |
| (Salih et al., 2020) | - Do you think smoking shisha will be less addictive than cigarette smoking? | Cigarettes vs. waterpipe |
| (Saravanan et al., 2019) | - Is shisha smoking more addictive than cigarettes? | Cigarettes vs. waterpipe |
| (Ward et al., 2007) | - How do you compare the addictive effects of smoking tobacco using a waterpipe versus smoking cigarettes? | Cigarettes vs. waterpipe |
| **Across Different Products (n = 4)** | | |
| (Grassi et al., 2012) | - Nicotine is as addictive as heroin or cocaine | Nicotine vs. heroin/cocaine |
| (Palinkas et al., 1993) | - Tobacco is not as addictive as other drugs | Tobacco vs. other drugs |
| (Smith et al., 2011) | - Cigarette smoking is as addictive as cocaine | Cigarettes vs. cocaine |
| (Yan et al., 2008) | - Smoking is as addictive as using heroin | Tobacco vs. heroin |

Abdel-Qader, D. H., & Al Meslamani, A. Z. (2021). Knowledge and beliefs of Jordanian community toward e-cigarettes: A national survey. *Journal of Community Health*, *46*(3), Article 3. https://doi.org/10.1007/s10900-020-00896-8

Abughosh, S., Wu, I.-H., Peters, R. J., Hawari, F., & Essien, E. J. (2012). Ethnicity and waterpipe smoking among US students. *The International Journal of Tuberculosis and Lung Disease*, *16*(11), 1551–1557. https://doi.org/10.5588/ijtld.12.0152

Agaku, I. T., & Filippidis, F. T. (2014). Prevalence, determinants and impact of unawareness about the health consequences of tobacco use among 17,929 school personnel in 29 African countries. *BMJ Open*, *4*(8), Article 8. https://doi.org/10.1136/bmjopen-2014-005837

Aghar, H., El-Khoury, N., Reda, M., Hamadeh, W., Krayem, H., Mansour, M., Raouf, H., & Jaffa, M. A. (2020). Knowledge and attitudes towards E-cigarette use in Lebanon and their associated factors. *BMC Public Health*, *20*(1), Article 1. https://doi.org/10.1186/s12889-020-8381-x

Alam, M. M., Ward, K. D., Bahelah, R., Kalan, M. E., Asfar, T., Eissenberg, T., & Maziak, W. (2020). The Syrian Center for Tobacco Studies-13 (SCTS-13): Psychometric evaluation of a waterpipe-specific nicotine dependence instrument. *Drug and Alcohol Dependence*, *215*, 108192. https://doi.org/10.1016/j.drugalcdep.2020.108192

Alizaga, N. M., Hartman-Filson, M., Elser, H., Halpern-Felsher, B., & Vijayaraghavan, M. (2020). Alternative flavored and unflavored tobacco product use and cigarette quit attempts among current smokers experiencing homelessness. *Addictive Behaviors Reports*, *12*, 8. APA PsycInfo®. https://doi.org/10.1016/j.abrep.2020.100280

Al-Naggar, R. A., Bobryshev, Y. V., & Anil, S. (2014). Pattern of shisha and cigarette smoking in the general population in Malaysia. *Asian Pacific Journal of Cancer Prevention: APJCP*, *15*(24), Article 24. https://doi.org/10.7314/apjcp.2014.15.24.10841

Al-Naggar, R. A., & Saghir, F. S. A. (2011). Water pipe (shisha) smoking and associated factors among Malaysian university students. *Asian Pacific Journal of Cancer Prevention: APJCP*, *12*(11), Article 11.

Amin, T. T., Amr, M. A. M., Zaza, B. O., & Suleman, W. (2010). Harm perception, attitudes and predictors of waterpipe (shisha) smoking among secondary school adolescents in Al-Hassa, Saudi Arabia. *Asian Pacific Journal of Cancer Prevention: APJCP*, *11*(2), Article 2.

Anjum, Q., Ahmed, F., & Ashfaq, T. (2008). Knowledge, attitude and perception of water pipe smoking (Shisha) among adolescents aged 14-19 years. *JPMA. The Journal of the Pakistan Medical Association*, *58*(6), Article 6.

Aqeeli, A. A., Makeen, A. M., Al Bahhawi, T., Ryani, M. A., Bahri, A. A., Alqassim, A. Y., & El-Setouhy, M. (2022). Awareness, knowledge and perception of electronic cigarettes among undergraduate students in Jazan Region, Saudi Arabia. *Health & Social Care in the Community*, *30*(2), Article 2. https://doi.org/10.1111/hsc.13184

Arnett, J. J. (2000). Optimistic bias in adolescent and adult smokers and nonsmokers. *Addictive Behaviors*, *25*(4), Article 4. https://doi.org/10.1016/s0306-4603(99)00072-6

Aryal, U. R., & Lohani, S. P. (2011). Perceived risk of cigarette smoking among college students. *Journal of Nepal Health Research Council*, *9*(2), Article 2.

Asfar, T., Ward, K. D., Eissenberg, T., & Maziak, W. (2005). Comparison of patterns of use, beliefs, and attitudes related to waterpipe between beginning and established smokers. *BMC Public Health*, *5*, 19. https://doi.org/10.1186/1471-2458-5-19

Ashley, M. J., Cohen, J., Bull, S., Ferrence, R., Poland, B., Pederson, L., & Gao, J. (2000). Knowledge about tobacco and attitudes toward tobacco control: How different are smokers and nonsmokers? *Canadian Journal of Public Health*, *91*(5), 376–380. https://doi.org/10.1007/BF03404811

Ashley, M. J., Cohen, J., & Ferrence, R. (2001). “Light” and “mild” cigarettes: Who smokes them? Are they being misled? *Canadian Journal of Public Health = Revue Canadienne De Sante Publique*, *92*(6), Article 6.

Azab, M., Khabour, O. F., Alkaraki, A. K., Eissenberg, T., Alzoubi, K. H., & Primack, B. A. (2010). Water pipe tobacco smoking among university students in Jordan. *Nicotine & Tobacco Research: Official Journal of the Society for Research on Nicotine and Tobacco*, *12*(6), Article 6. https://doi.org/10.1093/ntr/ntq055

Azab, M., Khabour, O. F., Alzoubi, K. H., Anabtawi, M. M., Quttina, M., Khader, Y., & Eissenberg, T. (2013). Exposure of pregnant women to waterpipe and cigarette smoke. *Nicotine & Tobacco Research: Official Journal of the Society for Research on Nicotine and Tobacco*, *15*(1), Article 1. https://doi.org/10.1093/ntr/nts119

Baig, M., Bakarman, M. A., Gazzaz, Z. J., Khabaz, M. N., Ahmed, T. J., Qureshi, I. A., Hussain, M. B., Alzahrani, A. H., AlShehri, A. A., Basendwah, M. A., Altherwi, F. B., & AlShehri, F. M. (2016). Reasons and motivations for cigarette smoking and barriers against quitting among a sample of young people in Jeddah, Saudi Arabia. *Asian Pacific Journal of Cancer Prevention: APJCP*, *17*(7), Article 7.

Balmford, J., & Borland, R. (2008). What does it mean to want to quit? *Drug and Alcohol Review*, *27*(1), Article 1. https://doi.org/10.1080/09595230701710829

Barker, J. O., Kelley, D. E., Noar, S. M., Reboussin, B. A., Cornacchione Ross, J., & Sutfin, E. L. (2019). E-cigarette outcome expectancies among nationally representative samples of adolescents and young adults. *Substance Use & Misuse*, *54*(12), Article 12. APA PsycInfo®. https://doi.org/10.1080/10826084.2019.1624773

Berg, C. J., Haardörfer, R., Wagener, T. L., Kegler, M. C., & Windle, M. (2018). Correlates of allowing alternative tobacco product or marijuana use in the homes of young adults. *Pediatrics*, *141*(Suppl 1), Article Suppl 1. https://doi.org/10.1542/peds.2017-1026E

Berg, C. J., Romm, K. F., Patterson, B., & Wysota, C. N. (2021). Heated tobacco product awareness, use, and perceptions in a sample of young adults in the United States. *Nicotine & Tobacco Research: Official Journal of the Society for Research on Nicotine and Tobacco*, *23*(11), Article 11. https://doi.org/10.1093/ntr/ntab058

Berg, C. J., Stratton, E., Schauer, G. L., Lewis, M., Wang, Y., Windle, M., & Kegler, M. (2015). Perceived harm, addictiveness, and social acceptability of tobacco products and marijuana among young adults: Marijuana, hookah, and electronic cigarettes win. *Substance Use & Misuse*, *50*(1), Article 1. https://doi.org/10.3109/10826084.2014.958857

Berg, M. B., Lin, L., White, M., & Alfonso-Barry, J. (2017). Attitudinal and behavioral differences between cigarette users who do and do not identify as “smokers.” *Journal of American College Health: J of ACH*, *65*(6), Article 6. https://doi.org/10.1080/07448481.2017.1312417

Berry, C., & Burton, S. (2019). Reduced-risk warnings versus the US FDA-mandated addiction warning: The effects of e-cigarette warning variations on health risk perceptions. *Nicotine & Tobacco Research: Official Journal of the Society for Research on Nicotine and Tobacco*, *21*(7), Article 7. https://doi.org/10.1093/ntr/nty177

Berry, C., Burton, S., & Howlett, E. (2017a). Are cigarette smokers’, E-cigarette users’, and dual users’ health-risk beliefs and responses to advertising influenced by addiction warnings and product type? *Nicotine & Tobacco Research*, *19*(10), Article 10. APA PsycInfo®. https://doi.org/10.1093/ntr/ntx075

Berry, C., Burton, S., & Howlett, E. (2017b). The impact of e-cigarette addiction warnings and health-related claims on consumers’ risk beliefs and use intentions. *Journal of Public Policy & Marketing*, *36*(1), Article 1. APA PsycInfo®. https://doi.org/10.1509/jppm.15.024

Blomqvist, J. (2012). Perceptions of addiction and recovery in Sweden: The influence of respondent characteristics. *Addiction Research & Theory*, *20*(5), Article 5. APA PsycInfo®. https://doi.org/10.3109/16066359.2012.664203

Borgan, S. M., Marhoon, Z. A., & Whitford, D. L. (2013). Beliefs and perceptions toward quitting waterpipe smoking among cafe waterpipe tobacco smokers in Bahrain. *Nicotine & Tobacco Research: Official Journal of the Society for Research on Nicotine and Tobacco*, *15*(11), Article 11. https://doi.org/10.1093/ntr/ntt064

Braun, R. E., Glassman, T., Wohlwend, J., Whewell, A., & Reindl, D. M. (2012). Hookah use among college students from a Midwest University. *Journal of Community Health*, *37*(2), Article 2. https://doi.org/10.1007/s10900-011-9444-9

Brennan, E., Gibson, L., Momjian, A., & Hornik, R. C. (2015). Are young people’s beliefs about menthol cigarettes associated with smoking-related intentions and behaviors? *Nicotine & Tobacco Research: Official Journal of the Society for Research on Nicotine and Tobacco*, *17*(1), Article 1. https://doi.org/10.1093/ntr/ntu134

Brownson, R. C., Jackson-Thompson, J., Wilkerson, J. C., Davis, J. R., Owens, N. W., & Fisher, E. B. (1992). Demographic and socioeconomic differences in beliefs about the health effects of smoking. *American Journal of Public Health*, *82*(1), Article 1. APA PsycInfo®. https://doi.org/10.2105/AJPH.82.1.99

Brożek, G., Jankowski, M., Zejda, J., Jarosińska, A., Idzik, A., & Bańka, P. (2017). E-smoking among students of medicine—Frequency, pattern and motivations. *Advances in Respiratory Medicine*, *85*(1), Article 1. https://doi.org/10.5603/ARM.2017.0003

Budd, G. M., & Preston, D. B. (2005). College students’ attitudes and beliefs about the consequences of smoking: Development and normative scores of a new scale. *Journal of the American Academy of Nurse Practitioners*, *13*(9), 421–427. https://doi.org/10.1111/j.1745-7599.2001.tb00061.x

Byron, M. J., Hall, M. G., King, J. L., Ribisl, K. M., & Brewer, N. T. (2019). Reducing nicotine without misleading the public: Descriptions of cigarette nicotine level and accuracy of perceptions about nicotine content, addictiveness, and risk. *Nicotine & Tobacco Research: Official Journal of the Society for Research on Nicotine and Tobacco*, *21*(Suppl 1), Article Suppl 1. https://doi.org/10.1093/ntr/ntz161

Calhoun, J. F. (1974). Attitudes toward the sale and use of drugs—A cross-sectional analysis of those who used drugs. *Journal of Youth and Adolescence*, *3*(1), 31–47. https://doi.org/10.1007/BF02215335

Camara-Medeiros, A., Diemert, L., O’Connor, S., Schwartz, R., Eissenberg, T., & Cohen, J. E. (2021). Perceived addiction to vaping among youth and young adult regular vapers. *Tobacco Control*, *30*(3), Article 3. https://doi.org/10.1136/tobaccocontrol-2019-055352

Case, K. R., Hinds, J. T., Creamer, M. R., Loukas, A., & Perry, C. L. (2020). Who is JUULing and why? An examination of young adult electronic nicotine delivery systems users. *The Journal of Adolescent Health: Official Publication of the Society for Adolescent Medicine*, *66*(1), Article 1. https://doi.org/10.1016/j.jadohealth.2019.05.030

Ceballos, N. A., Wiese, B., & Hovland, J. (2009). Gender, body image, and attitudes about tobacco in the United States–Mexico border region: Implications for individualized prevention and treatment efforts for adolescents and emerging adults. *Journal of Addictions Nursing*, *20*(2), Article 2. APA PsycInfo®. https://doi.org/10.1080/10884600902850103

Center for Disease Control and Prevention. (2015). *National Adult Tobacco Survey* [Dataset]. https://www.cdc.gov/tobacco/data_statistics/surveys/nats/pdfs/2013-2014-questionnaire-tag508.pdf

Chaaya, M., Jabbour, S., El-Roueiheb, Z., & Chemaitelly, H. (2004). Knowledge, attitudes, and practices of argileh (water pipe or hubble-bubble) and cigarette smoking among pregnant women in Lebanon. *Addictive Behaviors*, *29*(9), Article 9. https://doi.org/10.1016/j.addbeh.2004.04.008

Chaaya, M., Roueiheb, Z. E., Chemaitelly, H., Azar, G., Nasr, J., & Al-Sahab, B. (2004). Argileh smoking among university students: A new tobacco epidemic. *Nicotine & Tobacco Research*, *6*(3), Article 3. https://doi.org/10.1080/14622200410001696628

Chaiton, M., Cohen, J. E., Bondy, S. J., Selby, P., Brown, K. S., Ferrence, R., & Garcia, J. M. (2017). Perceived addiction as a predictor of smoking cessation among occasional smokers. *Journal of Smoking Cessation*, *12*(3), Article 3. APA PsycInfo®. https://doi.org/10.1017/jsc.2015.19

Chassin, L., Presson, C. C., Rose, J., & Sherman, S. J. (2007). What is addiction? Age-related differences in the meaning of addiction. *Drug and Alcohol Dependence*, *87*(1), Article 1. APA PsycInfo®. https://doi.org/10.1016/j.drugalcdep.2006.07.006

Chassin, L., Presson, C. C., Sherman, S. J., Seo, D.-C., & Macy, J. T. (2010). Implicit and explicit attitudes predict smoking cessation: Moderating effects of experienced failure to control smoking and plans to quit. *Psychology of Addictive Behaviors: Journal of the Society of Psychologists in Addictive Behaviors*, *24*(4), 670–679. https://doi.org/10.1037/a0021722

Chen, H.-L. (2009). Consumer risk perception and addictive consumption behavior. *Social Behavior and Personality: An International Journal*, *37*(6), Article 6. APA PsycInfo®. https://doi.org/10.2224/sbp.2009.37.6.767

Choi, K., Bestrashniy, J., & Forster, J. (2018). Trends in awareness, use of, and beliefs about electronic cigarette and snus among a longitudinal cohort of US midwest young adults. *Nicotine & Tobacco Research: Official Journal of the Society for Research on Nicotine and Tobacco*, *20*(2), Article 2. https://doi.org/10.1093/ntr/ntx042

Choi, K., & Forster, J. (2013a). Awareness, perceptions and use of snus among young adults from the upper Midwest region of the USA. *Tobacco Control*, *22*(6), Article 6. https://doi.org/10.1136/tobaccocontrol-2011-050383

Choi, K., & Forster, J. (2013b). Characteristics associated with awareness, perceptions, and use of electronic nicotine delivery systems among young US Midwestern adults. *American Journal of Public Health*, *103*(3), Article 3. https://doi.org/10.2105/AJPH.2012.300947

Choi, K., & Forster, J. L. (2014). Beliefs and experimentation with electronic cigarettes: A prospective analysis among young adults. *American Journal of Preventive Medicine*, *46*(2), Article 2. https://doi.org/10.1016/j.amepre.2013.10.007

Cooper, M., Loukas, A., Case, K. R., Marti, C. N., & Perry, C. L. (2018). A longitudinal study of risk perceptions and e-cigarette initiation among college students: Interactions with smoking status. *Drug and Alcohol Dependence*, *186*, 257–263. https://doi.org/10.1016/j.drugalcdep.2017.11.027

Cooper, M., Loukas, A., Harrell, M. B., & Perry, C. L. (2017). College students’ perceptions of risk and addictiveness of e-cigarettes and cigarettes. *Journal of American College Health: J of ACH*, *65*(2), Article 2. https://doi.org/10.1080/07448481.2016.1254638

Cooper, T. V., Taylor, T., Murray, A., DeBon, M. W., Vander Weg, M. W., Klesges, R. C., & Talcott, G. W. (2010). Differences between intermittent and light daily smokers in a population of U.S. military recruits. *Nicotine & Tobacco Research: Official Journal of the Society for Research on Nicotine and Tobacco*, *12*(5), Article 5. https://doi.org/10.1093/ntr/ntq025

Copeland, A. L., Peltier, M. R., & Waldo, K. (2017). Perceived risk and benefits of e-cigarette use among college students. *Addictive Behaviors*, *71*, 31–37. https://doi.org/10.1016/j.addbeh.2017.02.005

Correa, J. B., Brandon, K. O., Meltzer, L. R., Hoehn, H. J., Piñeiro, B., Brandon, T. H., & Simmons, V. N. (2018). Electronic cigarette use among patients with cancer: Reasons for use, beliefs, and patient-provider communication. *Psycho-Oncology*, *27*(7), Article 7. https://doi.org/10.1002/pon.4721

Creamer, M. R., Loukas, A., Li, X., Pasch, K. E., Case, K., Crook, B., & Perry, C. L. (2016). College students’ perceptions and knowledge of hookah use. *Drug and Alcohol Dependence*, *168*, 191–195. https://doi.org/10.1016/j.drugalcdep.2016.09.004

Cummings, K. M., Hyland, A., Bansal, M. A., & Giovino, G. A. (2004). What do Marlboro Lights smokers know about low-tar cigarettes? *Nicotine & Tobacco Research: Official Journal of the Society for Research on Nicotine and Tobacco*, *6 Suppl 3*, S323-332. https://doi.org/10.1080/14622200412331320725

Cummings, K. M., Hyland, A., Giovino, G., Hastrup, J., Bauer, J., & Bansal, M. (2004). Are smokers adequately informed about the health risks of smoking and medicinal nicotine? *Nicotine & Tobacco Research*, *6*(6), 333–340. https://doi.org/10.1080/14622200412331320734

Cummings, K. M., Sciandra, R., Gingrass, A., & Davis, R. (1991). What scientists funded by the tobacco industry believe about the hazards of cigarette smoking. *American Journal of Public Health*, *81*(7), Article 7. APA PsycInfo®. https://doi.org/10.2105/AJPH.81.7.894

Cummins, S., Leischow, S., Bailey, L., Bush, T., Wassum, K., Copeland, L., & Zhu, S.-H. (2016). Knowledge and beliefs about electronic cigarettes among quitline cessation staff. *Addictive Behaviors*, *60*, 78–83. https://doi.org/10.1016/j.addbeh.2016.03.031

Cunningham, J. A. (2012). Smokers and non-smokers differ in their beliefs about their addiction: Public health implications. *Canadian Journal of Public Health = Revue Canadienne De Sante Publique*, *103*(2), Article 2.

Dani, K. K., Oswal, K., Maudgal, S., & Saranath, D. (2015). Perception of young adults toward hookah use in Mumbai. *Indian Journal of Cancer*, *52*(4), Article 4. https://doi.org/10.4103/0019-509X.178384

Danishevski, K., Gilmore, A., & McKee, M. (2008). Public attitudes towards smoking and tobacco control policy in Russia. *Tobacco Control*, *17*(4), Article 4. https://doi.org/10.1136/tc.2008.025759

DeAtley, T., Denlinger-Apte, R. L., Cioe, P. A., Colby, S. M., Cassidy, R. N., Clark, M. A., Donny, E. C., & Tidey, J. W. (2020). Biopsychosocial mechanisms associated with tobacco use in smokers with and without serious mental illness. *Preventive Medicine*, *140*, 106190. https://doi.org/10.1016/j.ypmed.2020.106190

Denlinger-Apte, R. L., Joel, D. L., Strasser, A. A., & Donny, E. C. (2017). Low nicotine content descriptors reduce perceived health risks and positive cigarette ratings in participants using very low nicotine content cigarettes. *Nicotine & Tobacco Research*, *19*(10), 1149–1154.

Differding, M., Katz, S. J., Strayer, L. G., White, C., Strasser, A. A., Donny, E. C., Hatsukami, D. K., & Carroll, D. M. (2022). Educating the public on the health risks of very low nicotine content cigarettes: Results from a US-based convenience sample. *Nicotine & Tobacco Research*, *24*(6), 871–880. https://doi.org/10.1093/ntr/ntac010

DiFranza, J. R., Morello, P., & Gershenson, B. (2012). The retest reliability of nicotine dependence measures. *Addiction Research & Theory*, *20*(1), Article 1. APA PsycInfo®. https://doi.org/10.3109/16066359.2011.558956

Dixit, D., Herbst, E., & Das, S. (2021). E-cigarette use and perceptions among veterans receiving outpatient treatment in Veterans Affairs substance use and mental health clinics. *Military Medicine*, *186*(1–2), Article 1–2. APA PsycInfo®. https://doi.org/10.1093/milmed/usaa292

Edwards, S. A., Bondy, S. J., Kowgier, M., McDonald, P. W., & Cohen, Joanna E. (2010). Are occasional smokers a heterogeneous group? An exploratory study. *Nicotine & Tobacco Research*, *12*(12), 1195–1202. https://doi.org/10.1093/ntr/ntq168

Egnot, E., Jordan, K., & Elliott, J. O. (2017). Associations with resident physicians’ early adoption of electronic cigarettes for smoking cessation. *Postgraduate Medical Journal*, *93*(1100), 319–325. https://doi.org/10.1136/postgradmedj-2016-134058

Eiser, J. R., Sutton, S. R., & Wober, M. (1977). Smokers, non-smokers and the attribution of addiction. *The British Journal of Social and Clinical Psychology*, *16*(4), Article 4. https://doi.org/10.1111/j.2044-8260.1977.tb00239.x

Eiser, J. R., & Van der Pligt, J. (1986). “Sick” or “hooked”: Smokers’ perceptions of their addiction. *Addictive Behaviors*, *11*(1), Article 1. https://doi.org/10.1016/0306-4603(86)90003-1

Eiser, J. R., Van der Pligt, J., Raw, M., & Sutton, S. R. (1985). Trying to stop smoking: Effects of perceived addiction, attributions for failure, and expectancy of success. *Journal of Behavioral Medicine*, *8*(4), Article 4. APA PsycInfo®. https://doi.org/10.1007/BF00848367

Etter, J.-F. (2015). Explaining the effects of electronic cigarettes on craving for tobacco in recent quitters. *Drug and Alcohol Dependence*, *148*, 102–108. https://doi.org/10.1016/j.drugalcdep.2014.12.030

Etter, J.-F. (2016). Throat hit in users of the electronic cigarette: An exploratory study. *Psychology of Addictive Behaviors: Journal of the Society of Psychologists in Addictive Behaviors*, *30*(1), Article 1. https://doi.org/10.1037/adb0000137

Etter, J.-F., & Bullen, C. (2011). Electronic cigarette: Users profile, utilization, satisfaction and perceived efficacy. *Addiction (Abingdon, England)*, *106*(11), Article 11. https://doi.org/10.1111/j.1360-0443.2011.03505.x

Farrimond, H. (2017). A typology of vaping: Identifying differing beliefs, motivations for use, identity and political interest amongst e-cigarette users. *International Journal of Drug Policy*, *48*, 81–90. APA PsycInfo®. https://doi.org/10.1016/j.drugpo.2017.07.011

Fevrier, B., Nabors, L., Vidourek, R. A., & King, K. A. (2018). Hookah use among college students: Recent use, knowledge of health risks, attitude and reasons for use. *Journal of Community Health*, *43*(6), Article 6. https://doi.org/10.1007/s10900-018-0519-8

Galimov, A., Meza, L., Unger, J. B., Baezconde-Garbanati, L., Cruz, T. B., & Sussman, S. (2021). Vape shop employees: Do they act as smoking cessation counselors? *Nicotine & Tobacco Research: Official Journal of the Society for Research on Nicotine and Tobacco*, *23*(4), Article 4. https://doi.org/10.1093/ntr/ntaa218

Garbutt, J. M., Miller, W., Dodd, S., Bobenhouse, N., Sterkel, R., & Strunk, R. C. (2015). Parental use of electronic cigarettes. *Academic Pediatrics*, *15*(6), Article 6. https://doi.org/10.1016/j.acap.2015.06.013

Garey, L., Mayorga, N. A., Peraza, N., Smit, T., Nizio, P., Otto, M. W., & Zvolensky, M. J. (2019). Distinguishing characteristics of e-cigarette users who attempt and fail to quit: Dependence, perceptions, and affective vulnerability. *Journal of Studies on Alcohol and Drugs*, *80*(1), Article 1. APA PsycInfo®. https://doi.org/10.15288/jsad.2019.80.134

Garey, L., Smit, T., Mayorga, N. A., Peraza, N., Nizio, P., Otto, M. W., & Zvolensky, M. J. (2019). Differential effects of anxiety sensitivity on e-cigarettes processes: The importance of e-cigarette quit attempt history. *The American Journal on Addictions*, *28*(5), 390–397. https://doi.org/10.1111/ajad.12940

Gillies, P. A., Madeley, R. J., & Power, F. L. (1989). Why do pregnant women smoke? *Public Health*, *103*(5), Article 5. https://doi.org/10.1016/s0033-3506(89)80003-4

Gilpin, E. A., Emery, S., White, M. M., & Pierce, J. P. (2002). Does tobacco industry marketing of “light” cigarettes give smokers a rationale for postponing quitting? *Nicotine & Tobacco Research: Official Journal of the Society for Research on Nicotine and Tobacco*, *4 Suppl 2*, S147-155. https://doi.org/10.1080/1462220021000032870

Glasser, A. M., Barton, A., Rath, J., Simard, B., Rose, S. W., Hair, E., & Vallone, D. (2020). Perceptions of use patterns and health consequences associated with mentholated cigarettes among U.S. adults. *Health Education & Behavior: The Official Publication of the Society for Public Health Education*, *47*(2), Article 2. https://doi.org/10.1177/1090198119897608

Goniewicz, M. L., Lingas, E. O., & Hajek, P. (2013). Patterns of electronic cigarette use and user beliefs about their safety and benefits: An internet survey. *Drug and Alcohol Review*, *32*(2), Article 2. https://doi.org/10.1111/j.1465-3362.2012.00512.x

Gottlieb, J. C., Cohen, L. M., DeMarree, K. G., Treloar, H. R., & McCarthy, D. M. (2013). The development and psychometric evaluation of the Smokeless Tobacco Expectancies Scale (STES). *Psychological Assessment*, *25*(3), Article 3. APA PsycInfo®. https://doi.org/10.1037/a0032256

Gottlieb, N. H., Gingiss, P. L., & Weinstein, R. P. (1992). Attitudes, subjective norms and models of use for smokeless tobacco among college athletes: Implications for prevention and cessation programming. *Health Education Research*, *7*(3), 359–368. APA PsycInfo®. https://doi.org/10.1093/her/7.3.359

Grassi, M. C., Chiamulera, C., Baraldo, M., Culasso, F., Ferketich, A. K., Raupach, T., Patrono, C., & Nencini, P. (2012). Cigarette smoking knowledge and perceptions among students in four Italian medical schools. *Nicotine & Tobacco Research: Official Journal of the Society for Research on Nicotine and Tobacco*, *14*(9), Article 9. https://doi.org/10.1093/ntr/ntr330

Hakim, S., Chowdhury, M. A. B., & Uddin, M. J. (2018). Correlates of attempting to quit smoking among adults in Bangladesh. *Addictive Behaviors Reports*, *8*, 1–7. APA PsycInfo®. https://doi.org/10.1016/j.abrep.2018.04.002

Harrell, P. T., Marquinez, N. S., Correa, J. B., Meltzer, L. R., Unrod, M., Sutton, S. K., Simmons, V. N., & Brandon, T. H. (2015). Expectancies for cigarettes, e-cigarettes, and nicotine replacement therapies among e-cigarette users (aka vapers). *Nicotine & Tobacco Research: Official Journal of the Society for Research on Nicotine and Tobacco*, *17*(2), Article 2. https://doi.org/10.1093/ntr/ntu149

Harrell, P. T., Simmons, V. N., Piñeiro, B., Correa, J. B., Menzie, N. S., Meltzer, L. R., Unrod, M., & Brandon, T. H. (2015). E-cigarettes and expectancies: Why do some users keep smoking? *Addiction (Abingdon, England)*, *110*(11), Article 11. https://doi.org/10.1111/add.13043

Hatsukami, D. K., Heishman, S. J., Vogel, R. I., Denlinger, R. L., Roper-Batker, A. N., Mackowick, K. M., Jensen, J., Murphy, S. E., Thomas, B. F., & Donny, E. (2013). Dose-response effects of spectrum research cigarettes. *Nicotine & Tobacco Research*, *15*(6), 1113–1121. https://doi.org/10.1093/ntr/nts247

Hatsukami, D. K., Vogel, R. I., Severson, H. H., Jensen, J. A., & O’Connor, R. J. (2016). Perceived health risks of snus and medicinal nicotine products. *Nicotine & Tobacco Research: Official Journal of the Society for Research on Nicotine and Tobacco*, *18*(5), Article 5. https://doi.org/10.1093/ntr/ntv200

Havermans, A., Pennings, J. L. A., Hegger, I., Elling, J. M., de Vries, H., Pauwels, C. G. G. M., & Talhout, R. (2021). Awareness, use and perceptions of cigarillos, heated tobacco products and nicotine pouches: A survey among Dutch adolescents and adults. *Drug and Alcohol Dependence*, *229*(Pt B), Article Pt B. https://doi.org/10.1016/j.drugalcdep.2021.109136

Hawash, M., Mosleh, R., Jarrar, Y., Hanani, A., & Hajyousef, Y. (2022). The prevalence of water pipe smoking and perceptions on its addiction among university students in Palestine, Jordan, and Turkey. *Asian Pacific Journal of Cancer Prevention: APJCP*, *23*(4), Article 4. https://doi.org/10.31557/APJCP.2022.23.4.1247

Hefner, K. R., Sollazzo, A., Mullaney, S., Coker, K. L., & Sofuoglu, M. (2019). E-cigarettes, alcohol use, and mental health: Use and perceptions of e-cigarettes among college students, by alcohol use and mental health status. *Addictive Behaviors*, *91*, 12–20. https://doi.org/10.1016/j.addbeh.2018.10.040

Heinz, A. J., Giedgowd, G. E., Crane, N. A., Veilleux, J. C., Conrad, M., Braun, A. R., Olejarska, N. A., & Kassel, J. D. (2013). A comprehensive examination of hookah smoking in college students: Use patterns and contexts, social norms and attitudes, harm perception, psychological correlates and co-occurring substance use. *Addictive Behaviors*, *38*(11), Article 11. https://doi.org/10.1016/j.addbeh.2013.07.009

Hershberger, A. R., Karyadi, K. A., VanderVeen, J. D., & Cyders, M. A. (2017). Beliefs about the direct comparison of e-cigarettes and cigarettes. *Substance Use & Misuse*, *52*(8), Article 8. APA PsycInfo®. https://doi.org/10.1080/10826084.2016.1268628

Hoetger, C., Bono, R. S., Nicksic, N. E., Barnes, A. J., & Cobb, C. O. (2019). Influence of electronic cigarette characteristics on susceptibility, perceptions, and abuse liability indices among combustible tobacco cigarette smokers and non-smokers. *International Journal of Environmental Research and Public Health*, *16*(10), Article 10. https://doi.org/10.3390/ijerph16101825

Hughes, J. R., Keely, J. P., & Callas, P. W. (2005). Ever users versus never users of a “less risky” cigarette. *Psychology of Addictive Behaviors: Journal of the Society of Psychologists in Addictive Behaviors*, *19*(4), 439–442. https://doi.org/10.1037/0893-164X.19.4.439

Hughes, J. R., & Naud, S. (2016a). Abstinence expectancies and quit attempts. *Addictive Behaviors*, *63*, 93–96. https://doi.org/10.1016/j.addbeh.2016.07.009

Hughes, J. R., & Naud, S. (2016b). Perceived role of motivation and self-efficacy in smoking cessation: A secondary data analysis. *Addictive Behaviors*, *61*, 58–61. https://doi.org/10.1016/j.addbeh.2016.05.010

Janik-Koncewicz, K., Zatoński, T., Połtyn-Zaradna, K., Zatońska, K., Cedzyńska, M., Przewoźniak, K., & Wojtyła, A. (2012). An attempt to assess knowledge about tobacco dependence among students at the Medical University in Wroclaw. *Annals of Agricultural and Environmental Medicine: AAEM*, *19*(3), Article 3.

Jiang, N., Cleland, C. M., Wang, M. P., Kwong, A., Lai, V., & Lam, T. H. (2019). Perceptions and use of e-cigarettes among young adults in Hong Kong. *BMC Public Health*, *19*(1), Article 1. https://doi.org/10.1186/s12889-019-7464-z

Jongenelis, M. I., Kameron, C., Rudaizky, D., Slevin, T., & Pettigrew, S. (2019). Perceptions of the harm, addictiveness, and smoking cessation effectiveness of e-cigarettes among Australian young adults. *Addictive Behaviors*, *90*, 217–221. https://doi.org/10.1016/j.addbeh.2018.11.004

Kaufman, A. R., Mays, D., Koblitz, A. R., & Portnoy, D. B. (2014). Judgments, awareness, and the use of snus among adults in the United States. *Nicotine & Tobacco Research: Official Journal of the Society for Research on Nicotine and Tobacco*, *16*(10), Article 10. https://doi.org/10.1093/ntr/ntu116

Kimber, C., Frings, D., Cox, S., Albery, I., & Dawkins, L. (2018). The effects of the European e-cigarette health warnings and comparative health messages on non-smokers’ and smokers’ risk perceptions and behavioural intentions. *BMC Public Health*, *18*(1), 1259. https://doi.org/10.1186/s12889-018-6161-7

Kimber, C., Frings, D., Cox, S., Albery, I. P., & Dawkins, L. (2020). Communicating the relative health risks of E-cigarettes: An online experimental study exploring the effects of a comparative health message versus the EU nicotine addiction warnings on smokers’ and non-smokers’ risk perceptions and behavioural intentions. *Addictive Behaviors*, *101*, 106177. https://doi.org/10.1016/j.addbeh.2019.106177

Kolar, S. K., Rogers, B. G., & Hooper, M. W. (2014). Support for indoor bans on electronic cigarettes among current and former smokers. *International Journal of Environmental Research and Public Health*, *11*(12), 12174–12189. https://doi.org/10.3390/ijerph111212174

Konkolÿ Thege, B., Colman, I., el-Guebaly, N., Hodgins, D. C., Patten, S. B., Schopflocher, D., Wolfe, J., & Wild, T. C. (2015). Social judgments of behavioral versus substance-related addictions: A population-based study. *Addictive Behaviors*, *42*, 24–31. https://doi.org/10.1016/j.addbeh.2014.10.025

Konkolÿ Thege, B., Colman, I., El-guebaly, N., Hodgins, D. C., Patten, S. B., Schopflocher, D., Wolfe, J., & Wild, T. C. (2015). Substance-related and behavioural addiction problems: Two surveys of Canadian adults. *Addiction Research & Theory*, *23*(1), Article 1. APA PsycInfo®. https://doi.org/10.3109/16066359.2014.923408

Koski-Jännes, A., Hirschovits-Gerz, T., Pennonen, M., & Nyyssönen, M. (2012). Population, professional and client views on the dangerousness of addictions: Testing the familiarity hypothesis. *NAT Nordisk Alkohol & Narkotikatidskrift*, *29*(2), Article 2. APA PsycInfo®. https://doi.org/10.2478/v10199-012-0010-2

Landry, R. L., Groom, A. L., Vu, T.-H. T., Stokes, A. C., Berry, K. M., Kesh, A., Hart, J. L., Walker, K. L., Giachello, A. L., Sears, C. G., McGlasson, K. L., Tompkins, L. K., Mattingly, D. T., Robertson, R. M., & Payne, T. J. (2019). The role of flavors in vaping initiation and satisfaction among U.S. adults. *Addictive Behaviors*, *99*, 106077. https://doi.org/10.1016/j.addbeh.2019.106077

Lee, H.-Y., Lin, H.-C., Seo, D.-C., & Lohrmann, D. K. (2018). The effect of e-cigarette warning labels on college students’ perception of e-cigarettes and intention to use e-cigarettes. *Addictive Behaviors*, *76*, 106–112. https://doi.org/10.1016/j.addbeh.2017.07.033

Lenk, K. M., Chen, V., Bernat, D. H., Forster, J. L., & Rode, P. A. (2009). Characterizing and comparing young adult intermittent and daily smokers. *Substance Use & Misuse*, *44*(14), Article 14. https://doi.org/10.3109/10826080902864571

Levinson, A. H., Campo, S., Gascoigne, J., Jolly, O., Zakharyan, A., & Tran, Z. V. (2007). Smoking, but not smokers: Identity among college students who smoke cigarettes. *Nicotine & Tobacco Research: Official Journal of the Society for Research on Nicotine and Tobacco*, *9*(8), Article 8. https://doi.org/10.1080/14622200701484987

Li, V. C., Hu, J. H., Zhou, M. L., & Zheng, J. B. (1988). Behavioral aspects of cigarette smoking among industrial college men of Shanghai, China. *American Journal of Public Health*, *78*(12), 1550–1553. https://doi.org/10.2105/AJPH.78.12.1550

Lim, R., Ishler, K., Trapl, E., & Flocke, S. (2019). “Phantom smokers”: Young cigarillo users who do not identify as smokers. *Drug and Alcohol Dependence*, *204*, 107551. https://doi.org/10.1016/j.drugalcdep.2019.107551

Lipkus, I. M., Eissenberg, T., Schwartz-Bloom, R. D., Prokhorov, A. V., & Levy, J. (2011). Affecting perceptions of harm and addiction among college waterpipe tobacco smokers. *Nicotine & Tobacco Research: Official Journal of the Society for Research on Nicotine and Tobacco*, *13*(7), Article 7. https://doi.org/10.1093/ntr/ntr049

Lipkus, I. M., Eissenberg, T., Schwartz-Bloom, R. D., Prokhorov, A. V., & Levy, J. (2014). Relationships among factual and perceived knowledge of harms of waterpipe tobacco, perceived risk, and desire to quit among college users. *Journal of Health Psychology*, *19*(12), Article 12. https://doi.org/10.1177/1359105313494926

Lipkus, I. M., & Mays, D. (2018). Comparing harm beliefs and risk perceptions among young adult waterpipe tobacco smokers and nonsmokers: Implications for cessation and prevention. *Addictive Behaviors Reports*, *7*, 103–110. APA PsycInfo®. https://doi.org/10.1016/j.abrep.2018.03.003

Lipkus, I. M., Mays, D., & Tercyak, K. P. (2017). Characterizing young adults’ susceptibility to waterpipe tobacco use and their reactions to messages about product harms and addictiveness. *Nicotine & Tobacco Research*, *19*(10), Article 10. APA PsycInfo®.

Lipkus, I. M., & Noonan, D. (2017). Association between felt ambivalence and the desire to quit waterpipe use among college students. *Journal of Health Psychology*, *22*(14), Article 14. https://doi.org/10.1177/1359105316636948

Lipkus, I. M., & Sanders, C. (2021). A pilot study assessing reactions to educational videos on harm of waterpipe among young adults susceptible to waterpipe tobacco smoking. *Journal of Health Communication*, *26*(11), 743–752. https://doi.org/10.1080/10810730.2021.2000522

Lipkus, I. M., Schwartz-Bloom, R., Kelley, M. J., & Pan, W. (2015). A preliminary exploration of college smokers’ reactions to nicotine dependence genetic susceptibility feedback. *Nicotine & Tobacco Research*, *17*(3), 337–343. https://doi.org/10.1093/ntr/ntu155

Lopes-Costa, E., & Amato-Vealey, E. (2016). Identifying beliefs about smoking in patients with peripheral vascular disease. *Journal of Vascular Nursing: Official Publication of the Society for Peripheral Vascular Nursing*, *34*(4), Article 4. https://doi.org/10.1016/j.jvn.2016.07.003

Lund, I., & Scheffels, J. (2014). Perceptions of relative risk of disease and addiction from cigarettes and snus. *Psychology of Addictive Behaviors: Journal of the Society of Psychologists in Addictive Behaviors*, *28*(2), Article 2. https://doi.org/10.1037/a0032657

Lydon, D. M., Howard, M. C., Wilson, S. J., & Geier, C. F. (2016). The perceived causal structures of smoking: Smoker and non-smoker comparisons. *Journal of Health Psychology*, *21*(9), Article 9. APA PsycInfo®. https://doi.org/10.1177/1359105315569895

Lyna, P., McBride, C., Samsa, G., & Pollak, K. I. (2002). Exploring the association between perceived risks of smoking and benefits to quitting: Who does not see the link? *Addictive Behaviors*, *27*(2), Article 2. https://doi.org/10.1016/s0306-4603(01)00175-7

Lynam, I., Catley, D., Harris, K. J., Goggin, K., Berkley-Patton, J., & Thomas, J. (2012). African American smokers’ intention to use pharmacotherapy for cessation. *American Journal of Health Behavior*, *36*(5), 615–627. https://doi.org/10.5993/AJHB.36.5.4

Majeed, B. A., Nyman, A., Sterling, K. L., & Slovic, P. (2018). Little cigars and cigarillos: Affect and perceived relative harm among U.S. adults, 2015. *Addictive Behaviors*, *85*, 107–112. https://doi.org/10.1016/j.addbeh.2018.05.024

Majeed, B. A., Weaver, S. R., Gregory, K. R., Whitney, C. F., Slovic, P., Pechacek, T. F., & Eriksen, M. P. (2017). Changing perceptions of harm of e-cigarettes among U.S. adults, 2012-2015. *American Journal of Preventive Medicine*, *52*(3), Article 3. https://doi.org/10.1016/j.amepre.2016.08.039

Majek, P., Jankowski, M., Nowak, B., Macherski, M., Nowak, M., Gil, A., Nakiela, P., Lewicka, B., Lawson, J. A., Zejda, J. E., & Brożek, G. M. (2021). The frequency of use and harm perception of heated tobacco products (HTPs): The 2019 cross-sectional survey among medical students from Poland. *International Journal of Environmental Research and Public Health*, *18*(7), Article 7. https://doi.org/10.3390/ijerph18073381

Marin, B. V., Perez-Stable, E. J., Marin, G., Sabogal, F., & Otero-Sabogal, R. (1990). Attitudes and behaviors of Hispanic smokers: Implications for cessation interventions. *Health Education Quarterly*, *17*(3), 287–297. https://doi.org/10.1177/109019819001700305

Mark, K. S., Farquhar, B., Chisolm, M. S., Coleman-Cowger, V. H., & Terplan, M. (2015). Knowledge, attitudes, and practice of electronic cigarette use among pregnant women. *Journal of Addiction Medicine*, *9*(4), Article 4. https://doi.org/10.1097/ADM.0000000000000128

Martin, D. S. (1990). Physical dependence and attributions of addiction among cigarette smokers. *Addictive Behaviors*, *15*(1), Article 1. https://doi.org/10.1016/0306-4603(90)90008-l

Mayorga, N. A., Garey, L., Nizio, P., Buckner, J. D., & Zvolensky, M. J. (2020). The effects of cannabis use: A test among dual electronic and combustible cigarette users. *The American Journal on Addictions*, *29*(4), 287–294. https://doi.org/10.1111/ajad.13021

Mayorga, N. A., Garey, L., & Zvolensky, M. J. (2019). Differences in perceptions of e-cigarettes across daily and non-daily users. *Addictive Behaviors*, *90*, 415–420. https://doi.org/10.1016/j.addbeh.2018.12.004

Mayorga, N. A., Smit, T., Shepherd, J. M., Orr, M. F., Garey, L., & Zvolensky, M. J. (2021). Worry and e-cigarette cognition: The moderating role of sex. *Addictive Behaviors*, *112*, 106621. https://doi.org/10.1016/j.addbeh.2020.106621

Mays, D., Moran, M. B., Levy, D. T., & Niaura, R. S. (2016). The impact of health warning labels for Swedish snus advertisements on young adults’ snus perceptions and behavioral intentions. *Nicotine & Tobacco Research: Official Journal of the Society for Research on Nicotine and Tobacco*, *18*(5), 1371–1375. https://doi.org/10.1093/ntr/ntv140

Mays, D., Smith, C., Johnson, A. C., Tercyak, K. P., & Niaura, R. S. (2016). An experimental study of the effects of electronic cigarette warnings on young adult nonsmokers’ perceptions and behavioral intentions. *Tobacco Induced Diseases*, *14*(1), 17. https://doi.org/10.1186/s12971-016-0083-x

Mays, D., Tercyak, K. P., & Lipkus, I. M. (2016). The effects of brief waterpipe tobacco use harm and addiction education messages among young adult waterpipe tobacco users. *Nicotine & Tobacco Research: Official Journal of the Society for Research on Nicotine and Tobacco*, *18*(5), Article 5. https://doi.org/10.1093/ntr/ntv223

Mays, D., Villanti, A., Niaura, R. S., Lindblom, E. N., & Strasser, A. A. (2019). The effects of varying electronic cigarette warning label design features on attention, recall, and product perceptions among young adults. *Health Communication*, *34*(3), 317–324. https://doi.org/10.1080/10410236.2017.1372050

Maziak, W., Ward, K. D., & Eissenberg, T. (2004). Factors related to frequency of narghile (waterpipe) use: The first insights on tobacco dependence in narghile users. *Drug and Alcohol Dependence*, *76*(1), Article 1. https://doi.org/10.1016/j.drugalcdep.2004.04.007

Meijer, E., & Chavannes, N. H. (2021). Lacking willpower? A latent class analysis of healthcare providers’ perceptions of smokers’ responsibility for smoking. *Patient Education and Counseling*, *104*(3), Article 3. APA PsycInfo®. https://doi.org/10.1016/j.pec.2020.08.027

Melin, K., Conte-Schmidt, N., Martínez-Arroyo, K., Rosa-Pérez, K., Soto-Avilés, A. E., & Hernández-Muñoz, J. J. (2018). Knowledge and perceptions of e-cigarettes and the motivations for their use: Talking to smokers (e-cigarettes and/or conventional cigarettes) and non-smokers in Puerto Rico. *Puerto Rico Health Sciences Journal*, *37*(3), Article 3.

Mercincavage, M., Lochbuehler, K., Villanti, A. C., Wileyto, E. P., Audrain-McGovern, J., & Strasser, A. A. (2019). Examining risk perceptions among daily smokers naïve to reduced nicotine content cigarettes. *Nicotine & Tobacco Research: Official Journal of the Society for Research on Nicotine and Tobacco*, *21*(7), Article 7. https://doi.org/10.1093/ntr/nty082

Miller, M. B., Lechner, W. V., Meier, E., Tucker, R. P., & Wiener, J. L. (2014). Dual tobacco use among college students: Contexts of use, self-perceptions, and attitudes toward quitting. *Substance Use & Misuse*, *49*(6), Article 6. APA PsycInfo®. https://doi.org/10.3109/10826084.2013.863345

Minhas, H. M., & Rahman, A. (2009). Prevalence, patterns and knowledge of effects on health of smoking among medical students in Pakistan. *Eastern Mediterranean Health Journal = La Revue De Sante De La Mediterranee Orientale = Al-Majallah Al-Sihhiyah Li-Sharq Al-Mutawassit*, *15*(5), Article 5.

Morgan, J. C., & Cappella, J. N. (2021). Harm perceptions and beliefs about potential modified risk tobacco products. *International Journal of Environmental Research and Public Health*, *18*(2), Article 2. https://doi.org/10.3390/ijerph18020576

Morphett, K., Puljević, C., Borland, R., Carter, A., Hall, W., & Gartner, C. (2021). Attitudes towards a hypothetical ‘clean nicotine’ product and harm reduction among smokers. *International Journal of Drug Policy*, *88*, 7. APA PsycInfo®. https://doi.org/10.1016/j.drugpo.2020.103020

Morrell, H. E. R., Cohen, L. M., & Dempsey, J. P. (2008). Smoking prevalence and awareness among undergraduate and health care students. *The American Journal on Addictions*, *17*(3), Article 3. https://doi.org/10.1080/10550490802019899

Moss, T. P., & Bould, E. (2009). A Q-methodological investigation into the meanings of cigarette consumption. *Journal of Health Psychology*, *14*(1), Article 1. APA PsycInfo®. https://doi.org/10.1177/1359105308097941

Mostafa, A. (2020). Self-reported addiction to and perceived behavioural control of waterpipe tobacco smoking and its patterns in Egypt: Policy implications. *Eastern Mediterranean Health Journal = La Revue De Sante De La Mediterranee Orientale = Al-Majallah Al-Sihhiyah Li-Sharq Al-Mutawassit*, *26*(1), Article 1. https://doi.org/10.26719/2020.26.1.18

Moysidou, A., Farsalinos, K. E., Voudris, V., Merakou, K., Kourea, K., & Barbouni, A. (2016). Knowledge and perceptions about nicotine, nicotine replacement therapies and electronic cigarettes among healthcare professionals in Greece. *International Journal of Environmental Research and Public Health*, *13*(5), Article 5. https://doi.org/10.3390/ijerph13050514

Murphy-Hoefer, R., Alder, S., & Higbee, C. (2004). Perceptions about cigarette smoking and risks among college students. *Nicotine & Tobacco Research: Official Journal of the Society for Research on Nicotine and Tobacco*, *6 Suppl 3*, S371-374. https://doi.org/10.1080/14622200412331320770

Netemeyer, R. G., Andrews, J. C., & Burton, S. (2005). Effects of antismoking advertising—Based beliefs on adult smokers’ consideration of quitting. *American Journal of Public Health*, *95*(6), Article 6. https://doi.org/10.2105/AJPH.2004.050195

Nicksic, N. E., Snell, L. M., Rudy, A. K., Cobb, C. O., & Barnes, A. J. (2017). Tobacco marketing, e-cigarette susceptibility, and perceptions among adults. *American Journal of Health Behavior*, *41*(5), Article 5. https://doi.org/10.5993/AJHB.41.5.7

Noonan, D., & Patrick, M. E. (2013). Factors associated with perceptions of hookah addictiveness and harmfulness among young adults. *Substance Abuse*, *34*(1), Article 1. APA PsycInfo®. https://doi.org/10.1080/08897077.2012.718251

North, C., Li, X., Grossberg, L. A., & Loukas, A. (2021). A one year prospective examination of risk factors for pod-vape use among young adults. *Drug and Alcohol Dependence*, *229*(Pt B), Article Pt B. https://doi.org/10.1016/j.drugalcdep.2021.109141

O’Connor, R. J., Heckman, B. W., Adkison, S. E., Rees, V. W., Hatsukami, D. K., Bickel, W. K., & Cummings, K. M. (2016). Persistence and amplitude of cigarette demand in relation to quit intentions and attempts. *Psychopharmacology*, *233*(12), 2365–2371. https://doi.org/10.1007/s00213-016-4286-x

Okoli, C. T. C., Rayens, M. K., Wiggins, A. T., Ickes, M. J., Butler, K. M., & Hahn, E. J. (2016). Secondhand tobacco smoke exposure and susceptibility to smoking, perceived addiction, and psychobehavioral symptoms among college students. *Journal of American College Health: J of ACH*, *64*(2), Article 2. https://doi.org/10.1080/07448481.2015.1074240

Pacek, L. R., Kozink, R. V., Carson, C. E., & McClernon, F. J. (2021). Appeal, subjective effects, and relative reinforcing effects of JUUL that vary in flavor and nicotine content. *Experimental and Clinical Psychopharmacology*, *29*(3), 279–287. APA PsycInfo®. https://doi.org/10.1037/pha0000481

Pacek, L. R., Oliver, J. A., Sweitzer, M. M., & McClernon, F. J. (2019). Young adult dual combusted cigarette and e-cigarette users’ anticipated responses to a nicotine reduction policy and menthol ban in combusted cigarettes. *Drug and Alcohol Dependence*, *194*, 40–44. APA PsycInfo®. https://doi.org/10.1016/j.drugalcdep.2018.10.005

Pacek, L. R., Rass, O., & Johnson, M. W. (2017). Knowledge about nicotine among HIV-positive smokers: Implications for tobacco regulatory science policy. *Addictive Behaviors*, *65*, 81–86. https://doi.org/10.1016/j.addbeh.2016.10.008

Palinkas, L. A., Pierce, J., Rosbrook, B. P., Pickwell, S., Johnson, M., & Bal, D. G. (1993). Cigarette smoking behavior and beliefs of Hispanics in California. *American Journal of Preventive Medicine*, *9*(6), Article 6. APA PsycInfo®.

Patel, M., Czaplicki, L., Perks, S. N., Cuccia, A. F., Liu, M., Hair, E. C., Schillo, B. A., & Vallone, D. M. (2019). Parents’ awareness and perceptions of JUUL and other e-cigarettes. *American Journal of Preventive Medicine*, *57*(5), Article 5. https://doi.org/10.1016/j.amepre.2019.06.012

Pechacek, T. F., Nayak, P., Slovic, P., Weaver, S. R., Huang, J., & Eriksen, M. P. (2018). Reassessing the importance of “lost pleasure” associated with smoking cessation: Implications for social welfare and policy. *Tobacco Control*, *27*(e2), e143–e151. https://doi.org/10.1136/tobaccocontrol-2017-053734

Perks, S. N., Haardörfer, R., Windle, M., & Berg, C. J. (2019). Tobacco abstinence motives in young adult college students: Scale development and validation. *American Journal of Health Behavior*, *43*(3), Article 3. https://doi.org/10.5993/AJHB.43.3.2

Perman-Howe, P. R., Horton, M., Robson, D., McDermott, M. S., McNeill, A., & Brose, L. S. (2022). Harm perceptions of nicotine-containing products and associated sources of information in UK adults with and without mental ill health: A cross-sectional survey. *Addiction (Abingdon, England)*, *117*(3), Article 3. https://doi.org/10.1111/add.15657

Perski, O., Herd, N., West, R., & Brown, J. (2019). Perceived addiction to smoking and associations with motivation to stop, quit attempts and quitting success: A prospective study of English smokers. *Addictive Behaviors*, *90*, 306–311. https://doi.org/10.1016/j.addbeh.2018.11.030

Peters, E. N., Harrell, P. T., Hendricks, P. S., O’Grady, K. E., Pickworth, W. B., & Vocci, F. J. (2015). Electronic cigarettes in adults in outpatient substance use treatment: Awareness, perceptions, use, and reasons for use. *The American Journal on Addictions*, *24*(3), Article 3. https://doi.org/10.1111/ajad.12206

Phan, L., Mays, D., Tercyak, K. P., Johnson, A. C., Rehberg, K., & Lipkus, I. M. (2021). Initial development of the Hookah Smoker Scale: Assessing young adults’ mental schemas about hookah “smokers.” *Translational Behavioral Medicine*, *11*(1), Article 1. https://doi.org/10.1093/tbm/ibz155

Pillitteri, J. L., Shiffman, S., Sembower, M. A., Polster, M. R., & Curtin, G. M. (2020). Assessing comprehension and perceptions of modified-risk information for snus among adult current cigarette smokers, former tobacco users, and never tobacco users. *Addictive Behaviors Reports*, *11*, 9. APA PsycInfo®. https://doi.org/10.1016/j.abrep.2020.100254

Pokhrel, P., Fagan, P., Herzog, T. A., Chen, Q., Muranaka, N., Kehl, L., & Unger, J. B. (2016). E-cigarette advertising exposure and implicit attitudes among young adult non-smokers. *Drug and Alcohol Dependence*, *163*, 134–140. https://doi.org/10.1016/j.drugalcdep.2016.04.008

Popova, L., Majeed, B., Owusu, D., Spears, C. A., & Ashley, D. L. (2018). Who are the smokers who never plan to quit and what do they think about the risks of using tobacco products? *Addictive Behaviors*, *87*, 62–68. https://doi.org/10.1016/j.addbeh.2018.06.024

Presson, C. C., Chassin, L., & Sherman, S. J. (2002). Psychosocial antecedents of tobacco chipping. *Health Psychology: Official Journal of the Division of Health Psychology, American Psychological Association*, *21*(4), Article 4. https://doi.org/10.1037//0278-6133.21.4.384

Primack, B. A., Sidani, J., Agarwal, A. A., Shadel, W. G., Donny, E. C., & Eissenberg, T. E. (2008). Prevalence of and associations with waterpipe tobacco smoking among U.S. university students. *Annals of Behavioral Medicine: A Publication of the Society of Behavioral Medicine*, *36*(1), Article 1. https://doi.org/10.1007/s12160-008-9047-6

Queloz, S., & Etter, J.-F. (2021). A survey of users of the IQOS tobacco vaporizer: Perceived dependence and perceived effects on cigarette withdrawal symptoms. *Journal of Addictive Diseases*, *39*(2), Article 2. https://doi.org/10.1080/10550887.2020.1847994

Rahman, M. A., Joseph, B., & Nimmi, N. (2022). Electronic cigarettes or vaping: Are there any differences in the profiles, use and perceptions between a developed and a developing country? *International Journal of Environmental Research and Public Health*, *19*(3), Article 3. https://doi.org/10.3390/ijerph19031673

Rahman, M. A., Mahmood, M. A., Spurrier, N., Rahman, M., Choudhury, S. R., & Leeder, S. (2015). Why do Bangladeshi people use smokeless tobacco products? *Asia-Pacific Journal of Public Health*, *27*(2), Article 2. https://doi.org/10.1177/1010539512446957

Rass, O., Pacek, L. R., Johnson, P. S., & Johnson, M. W. (2015). Characterizing use patterns and perceptions of relative harm in dual users of electronic and tobacco cigarettes. *Experimental and Clinical Psychopharmacology*, *23*(6), Article 6. https://doi.org/10.1037/pha0000050

Rath, J. M., Greenberg, M., Pitzer, L., Emelle, B., Green, M., Liu, S. M., Willett, J., Rose, S. W., Hair, E. C., & Vallone, D. (2018). The association between menthol perceptions and support for a policy ban among US smokers. *Ethnicity & Disease*, *28*(3), Article 3. https://doi.org/10.18865/ed.28.3.177

Reddy, P., Meyer-Weitz, A., & Yach, D. (1996). Smoking status, knowledge of health effects and attitudes towards tobacco control in South Africa. *South African Medical Journal = Suid-Afrikaanse Tydskrif Vir Geneeskunde*, *86*(11), Article 11.

Roberts, M. E., & Ferketich, A. K. (2020). Hookah susceptibility and transitions over the first year of college. *Journal of Studies on Alcohol and Drugs*, *81*(2), Article 2. APA PsycInfo®. https://doi.org/10.15288/jsad.2020.81.195

Roys, M. R., Peltier, M. R., Stewart, S. A., Waters, A. F., Waldo, K. M., & Copeland, A. L. (2020). The association between problematic alcohol use, risk perceptions, and e-cigarette use. *The American Journal of Drug and Alcohol Abuse*, *46*(2), Article 2. APA PsycInfo®. https://doi.org/10.1080/00952990.2019.1654486

Sabogal, F., Otero-Sabogal, R., Pérez-Stable, E. J., Marín, B. V., & Marin, G. (1989). Perceived self-efficacy to avoid cigarette smoking and addiction: Differences between Hispanics and non-Hispanic Whites. *Hispanic Journal of Behavioral Sciences*, *11*(2), Article 2. APA PsycInfo®. https://doi.org/10.1177/07399863890112003

Sadava, S. W., & Weithe, H. (1985). Maintenance and attributions about smoking among smokers, nonsmokers, and ex-smokers. *International Journal of the Addictions*, *20*(10), Article 10. APA PsycInfo®. https://doi.org/10.3109/10826088509047244

Saddichha, S., Rekha, D. P., Patil, B. K., Murthy, P., Benegal, V., & Isaac, M. K. (2010). Knowledge, attitude and practices of Indian dental surgeons towards tobacco control: Advances towards prevention. *Asian Pacific Journal of Cancer Prevention: APJCP*, *11*(4), Article 4.

Salih, S., Shaban, S., Athwani, Z., Alyahyawi, F., Alharbi, S., Ageeli, F., Hakami, A., Ageeli, A., Jubran, O., & Sahloli, S. (2020). Prevalence, predictors, and characteristics of waterpipe smoking among Jazan University students in Saudi Arabia: A cross-sectional study. *Annals of Global Health*, *86*(1), Article 1. https://doi.org/10.5334/aogh.2912

Saravanan, C., Attlee, A., & Sulaiman, N. (2019). A cross sectional study on knowledge, beliefs and psychosocial predictors of shisha smoking among university students in Sharjah, United Arab Emirates. *Asian Pacific Journal of Cancer Prevention: APJCP*, *20*(3), Article 3. https://doi.org/10.31557/APJCP.2019.20.3.903

Sarfraz, M., Rahim Khan, H. A., Urooba, A., Manan, Z., Irfan, O., Nadeem, R., Baqir, H., Farooq, S., Khan, Z., Khan, J. A., & Saleem, S. (2018). Awareness, use and perceptions about E-cigarettes among adult smokers in Karachi, Pakistan. *JPMA. The Journal of the Pakistan Medical Association*, *68*(1), Article 1.

Schippers, G. M., & Cox, W. M. (1994). Problem perception and addictive behaviors among Dutch and American college students. *Drugs: Education, Prevention & Policy*, *1*(1), Article 1. APA PsycInfo®. https://doi.org/10.3109/09687639409028533

Scott, K. A., Mason, M. J., & Mason, J. D. (2015). I’m not a smoker: Constructing protected prototypes for risk behavior. *Journal of Business Research*, *68*(10), Article 10. APA PsycInfo®. https://doi.org/10.1016/j.jbusres.2015.03.021

Seigers, D. K., & Terry, C. P. (2011). Perceptions of risk among college smokers: Relationships to smoking status. *Addiction Research & Theory*, *19*(6), Article 6. APA PsycInfo®. https://doi.org/10.3109/16066359.2010.545155

Sendzik, T., McDonald, P. W., Brown, K. S., Hammond, D., & Ferrence, R. (2011). Planned quit attempts among Ontario smokers: Impact on abstinence. *Addiction (Abingdon, England)*, *106*(11), Article 11. https://doi.org/10.1111/j.1360-0443.2011.03498.x

Seng, S., Otachi, J. K., & Okoli, C. T. C. (2020). Reasons for tobacco use and perceived tobacco-related health risks in an inpatient psychiatric population. *Issues in Mental Health Nursing*, *41*(2), 161–167. APA PsycInfo®. https://doi.org/10.1080/01612840.2019.1630533

Shadel, W. G., Lerman, C., Cappella, J., Strasser, A. A., Pinto, A., & Hornik, R. (2006). Evaluating smokers’ reactions to advertising for new lower nicotine quest cigarettes. *Psychology of Addictive Behaviors: Journal of the Society of Psychologists in Addictive Behaviors*, *20*(1), 80–84. https://doi.org/10.1037/0893-164X.20.1.80

Siqués, P., Brito, J., Muñoz, C., Pasten, P., Zavala, P., & Vergara, J. (2006). Prevalence and characteristics of smoking in primary healthcare workers in Iquique, Chile. *Public Health*, *120*(7), Article 7. https://doi.org/10.1016/j.puhe.2006.01.008

Smit, T., Chavez, J., Garey, L., Mayorga, N. A., Rogers, A. H., & Zvolensky, M. J. (2022). Pain interference and cognitive processes of e-cigarette use: The differential effect of sex. *Experimental and Clinical Psychopharmacology*, *30*(2), 132–140. https://doi.org/10.1037/pha0000393

Smith, P., Bansal-Travers, M., O’Connor, R., Brown, A., Banthin, C., Guardino-Colket, S., & Cummings, K. M. (2011). Correcting over 50 years of tobacco industry misinformation. *American Journal of Preventive Medicine*, *40*(6), Article 6. https://doi.org/10.1016/j.amepre.2011.01.020

Smith-Simone, S., Maziak, W., Ward, K. D., & Eissenberg, T. (2008). Waterpipe tobacco smoking: Knowledge, attitudes, beliefs, and behavior in two U.S. samples. *Nicotine & Tobacco Research*, *10*(2), Article 2. APA PsycInfo®. https://doi.org/10.1080/14622200701825023

Smith-Simone, S. Y., Curbow, B. A., & Stillman, F. A. (2008). Differing psychosocial risk profiles of college freshmen waterpipe, cigar, and cigarette smokers. *Addictive Behaviors*, *33*(12), Article 12. https://doi.org/10.1016/j.addbeh.2008.07.017

Stearns, A. E., Spivak, A. L., & Givel, M. S. (2012). Behind the smokescreen: Native American tobacco use in Oklahoma. *International Quarterly of Community Health Education*, *33*(3), Article 3. https://doi.org/10.2190/IQ.33.3.f

Stein, M. D., Caviness, C. M., Grimone, K., Audet, D., Borges, A., & Anderson, B. J. (2015). E-cigarette knowledge, attitudes, and use in opioid dependent smokers. *Journal of Substance Abuse Treatment*, *52*, 73–77. APA PsycInfo®. https://doi.org/10.1016/j.jsat.2014.11.002

Sterling, K. L., Majeed, B. A., Nyman, A., & Eriksen, M. (2017). Risk perceptions of little cigar and cigarillo smoking among adult current cigarette smokers. *Nicotine & Tobacco Research: Official Journal of the Society for Research on Nicotine and Tobacco*, *19*(11), Article 11. https://doi.org/10.1093/ntr/ntw244

Stippekohl, B., Winkler, M. H., Walter, B., Kagerer, S., Mucha, R. F., Pauli, P., Vaitl, D., & Stark, R. (2012). Neural responses to smoking stimuli are influenced by smokers’ attitudes towards their own smoking behavior. *PLoS ONE*, *7*(11), e46782. https://doi.org/10.1371/journal.pone.0046782

Tan, A. S. L., Lee, C., & Bigman, C. A. (2016). Comparison of beliefs about e-cigarettes’ harms and benefits among never users and ever users of e-cigarettes. *Drug and Alcohol Dependence*, *158*, 67–75. https://doi.org/10.1016/j.drugalcdep.2015.11.003

Tercyak, K. P., Beville, K. W., Walker, L. R., Prahlad, S., Cogen, F. R., Sobel, D. O., & Streisand, R. (2005). Health attitudes, beliefs, and risk behaviors among adolescents and young adults with Type 1 diabetes. *Children’s Health Care*, *34*(3), Article 3. APA PsycInfo®. https://doi.org/10.1207/s15326888chc3403_1

Thawal, V. P., Tzelepis, F., Ahmadi, S., Palazzi, K., & Paul, C. (2022). Addiction perceptions among users of smokeless or combustible tobacco attending a tertiary care hospital in India. *Drug and Alcohol Review*, *41*(5), 1184–1194. https://doi.org/10.1111/dar.13440

Torchalla, I., Okoli, C. T. C., Malchy, L., & Johnson, J. L. (2011). Nicotine dependence and gender differences in smokers accessing community mental health services: Gender differences in smoking among individuals with SPMI. *Journal of Psychiatric and Mental Health Nursing*, *18*(4), 349–358. https://doi.org/10.1111/j.1365-2850.2010.01674.x

Trumbo, C. W. (2018). Influence of risk perception on attitudes and norms regarding electronic cigarettes. *Risk Analysis*, *38*(5), Article 5. APA PsycInfo®. https://doi.org/10.1111/risa.12918

United States Department of Health and Human Services, National Institutes of Health, National Institute on Drug Abuse, Food and Drug Administration, & Center for Tobacco Products. (2021). *Population Assessment of Tobacco and Health (PATH) Study [United States] Public-Use Files.* [Dataset]. https://doi.org/10.3886/ICPSR36498.vXX

Vander Martin, R., Cummings, S. R., & Coates, T. J. (1990). Ethnicity and smoking: Differences in White, Black, Hispanic, and Asian medical patients who smoke. *American Journal of Preventive Medicine*, *6*(4), Article 4.

Vigna-Taglianti, F., Alesina, M., Damjanović, L., Mehanović, E., Akanidomo, I., Pwajok, J., Prichard, G., van der Kreeft, P., Virk, H. K., & Unplugged Nigeria Coordination Group. (2019). Knowledge, attitudes and behaviours on tobacco, alcohol and other drugs among Nigerian secondary school students: Differences by geopolitical zones. *Drug and Alcohol Review*, *38*(6), Article 6. https://doi.org/10.1111/dar.12974

Villanti, A. C., Naud, S., West, J. C., Pearson, J. L., Wackowski, O. A., Hair, E., Rath, J. M., & Niaura, R. S. (2019). Latent classes of nicotine beliefs correlate with perceived susceptibility and severity of nicotine and tobacco products in US young adults. *Nicotine & Tobacco Research: Official Journal of the Society for Research on Nicotine and Tobacco*, *21*(Suppl 1), Article Suppl 1. https://doi.org/10.1093/ntr/ntz156

Villanti, A. C., Naud, S., West, J. C., Pearson, J. L., Wackowski, O. A., Niaura, R. S., Hair, E., & Rath, J. M. (2019). Prevalence and correlates of nicotine and nicotine product perceptions in U.S. young adults, 2016. *Addictive Behaviors*, *98*, 106020. https://doi.org/10.1016/j.addbeh.2019.06.009

Vogel, E. A., Henriksen, L., Schleicher, N. C., & Prochaska, J. J. (2021). Young people’s e-cigarette risk perceptions, policy attitudes, and past-month nicotine vaping in 30 U.S. cities. *Drug and Alcohol Dependence*, *229*(Pt A), Article Pt A. https://doi.org/10.1016/j.drugalcdep.2021.109122

Vu, T.-H. T., Hart, J. L., Groom, A., Landry, R. L., Walker, K. L., Giachello, A. L., Tompkins, L., Ma, J. Z., Kesh, A., Robertson, R. M., & Payne, T. J. (2019). Age differences in electronic nicotine delivery systems (ENDS) usage motivations and behaviors, perceived health benefit, and intention to quit. *Addictive Behaviors*, *98*, 106054. https://doi.org/10.1016/j.addbeh.2019.106054

Wackowski, O. A., Sontag, J. M., Hammond, D., O’Connor, R. J., Ohman-Strickland, P. A., Strasser, A. A., Villanti, A. C., & Delnevo, C. D. (2019). The impact of e-cigarette warnings, warning themes and inclusion of relative harm statements on young adults’ e-cigarette perceptions and use intentions. *International Journal of Environmental Research and Public Health*, *16*(2), Article 2. https://doi.org/10.3390/ijerph16020184

Wainwright, K., Perrotte, J. K., Bibriescas, N., Baumann, M. R., & Garza, R. T. (2019). Smoking expectancies and health perceptions: An analysis of Hispanic subgroups. *Addictive Behaviors*, *98*, 106008. https://doi.org/10.1016/j.addbeh.2019.05.032

Ward, K. D., Eissenberg, T., Gray, J. N., Srinivas, V., Wilson, N., & Maziak, W. (2007). Characteristics of U.S. waterpipe users: A preliminary report. *Nicotine & Tobacco Research: Official Journal of the Society for Research on Nicotine and Tobacco*, *9*(12), Article 12. https://doi.org/10.1080/14622200701705019

Ward, K. D., Hammal, F., VanderWeg, M. W., Eissenberg, T., Asfar, T., Rastam, S., & Maziak, W. (2005). Are waterpipe users interested in quitting? *Nicotine & Tobacco Research*, *7*(1), Article 1. APA PsycInfo®. https://doi.org/10.1080/14622200412331328402

Waters, E. A., Janssen, E., Kaufman, A. R., Peterson, L. M., Muscanell, N. L., Guadagno, R. E., & Stock, M. L. (2016). The relationship between young adult smokers’ beliefs about nicotine addiction and smoking-related affect and cognitions. *Journal of Cancer Education: The Official Journal of the American Association for Cancer Education*, *31*(2), Article 2. https://doi.org/10.1007/s13187-015-0819-y

Webb Hooper, M., & Kolar, S. K. (2017). Racial/ethnic differences in electronic cigarette knowledge, social norms, and risk perceptions among current and former smokers. *Addictive Behaviors*, *67*, 86–91. https://doi.org/10.1016/j.addbeh.2016.12.013

Weinstein, N. D., Slovic, P., & Gibson, G. (2004). Accuracy and optimism in smokers’ beliefs about quitting. *Nicotine & Tobacco Research: Official Journal of the Society for Research on Nicotine and Tobacco*, *6 Suppl 3*, S375-380. https://doi.org/10.1080/14622200412331320789

West, R., & Hargreaves, M. (1995). Factors associated with smoking in student nurses. *Psychology & Health*, *10*(3), Article 3. APA PsycInfo®. https://doi.org/10.1080/08870449508401949

Wilson, S., Partos, T., McNeill, A., & Brose, L. S. (2019). Harm perceptions of e-cigarettes and other nicotine products in a UK sample. *Addiction (Abingdon, England)*, *114*(5), Article 5. https://doi.org/10.1111/add.14502

Wolfson, M., Pockey, J. R., Reboussin, B. A., Sutfin, E. L., Egan, K. L., Wagoner, K. G., & Spangler, J. G. (2014). First-year college students’ interest in trying dissolvable tobacco products. *Drug and Alcohol Dependence*, *134*, 309–313. https://doi.org/10.1016/j.drugalcdep.2013.10.025

Yan, J., Xiao, S., Ouyang, D., Jiang, D., He, C., & Yi, S. (2008). Smoking behavior, knowledge, attitudes and practice among health care providers in Changsha City, China. *Nicotine & Tobacco Research: Official Journal of the Society for Research on Nicotine and Tobacco*, *10*(4), Article 4. https://doi.org/10.1080/14622200801901930

Yang, B., Owusu, D., & Popova, L. (2019a). Effects of a nicotine fact sheet on perceived risk of nicotine and e-cigarettes and intentions to seek information about and use e-cigarettes. *International Journal of Environmental Research and Public Health*, *17*(1), Article 1. https://doi.org/10.3390/ijerph17010131

Yang, B., Owusu, D., & Popova, L. (2019b). Testing messages about comparative risk of electronic cigarettes and combusted cigarettes. *Tobacco Control*, *28*(4), 440–448. https://doi.org/10.1136/tobaccocontrol-2018-054404

Yang, B., & Popova, L. (2020). Communicating risk differences between electronic and combusted cigarettes: The role of the FDA-mandated addiction warning and a nicotine fact sheet. *Tobacco Control*, *29*(6), Article 6. https://doi.org/10.1136/tobaccocontrol-2019-055204

Yang, B., Spears, C. A., & Popova, L. (2019). Psychological distress and responses to comparative risk messages about electronic and combusted cigarettes. *Addictive Behaviors*, *91*, 141–148. https://doi.org/10.1016/j.addbeh.2018.11.025

Yates, E. A., Dubray, J., Schwartz, R., Kirst, M., Lacombe-Duncan, A., Suwal, J., & Hatcher, J. (2014). Patterns of cigarillo use among Canadian young adults in two urban settings. *Canadian Journal of Public Health = Revue Canadienne De Sante Publique*, *105*(1), Article 1. https://doi.org/10.17269/cjph.105.3879

Yel, D., Bui, A., Job, J. S., Knutsen, S., & Singh, P. N. (2013). Beliefs about tobacco, health, and addiction among adults in Cambodia: Findings from a national survey. *Journal of Religion and Health*, *52*(3), Article 3. https://doi.org/10.1007/s10943-011-9537-x

Yunus, M., & Khan, Z. (1997). A baseline study of tobacco use among the staff of Aligarh Muslim University, Aligarh, India. *Journal of the Royal Society of Health*, *117*(6), Article 6. https://doi.org/10.1177/146642409711700606

Zgliczyński, W. S., Jankowski, M., Rostkowska, O., Gujski, M., Wierzba, W., & Pinkas, J. (2019). Knowledge and beliefs of e-cigarettes among physicians in Poland. *Medical Science Monitor: International Medical Journal of Experimental and Clinical Research*, *25*, 6322–6330. https://doi.org/10.12659/MSM.916920

Zinser, M. C., Pampel, F. C., & Flores, E. (2011). Distinct beliefs, attitudes, and experiences of Latino smokers: Relevance for cessation interventions. *American Journal of Health Promotion: AJHP*, *25*(5 Suppl), Article 5 Suppl. https://doi.org/10.4278/ajhp.100616-QUAN-200

Zvolensky, M. J., D’Souza, J., Garey, L., Alfano, C. A., Mayorga, N. A., Peraza, N., & Gallagher, M. W. (2020). Subjective sleep quality and electronic cigarette dependence, perceived risks of use, and perceptions about quitting electronic cigarettes. *Addictive Behaviors*, *102*, 106199. https://doi.org/10.1016/j.addbeh.2019.106199

Zvolensky, M. J., Garey, L., Mayorga, N. A., Rogers, A. H., Orr, M. F., Ditre, J. W., & Peraza, N. (2019). Current pain severity and electronic cigarettes: An initial empirical investigation. *Journal of Behavioral Medicine*, *42*(3), 461–468. https://doi.org/10.1007/s10865-018-9995-7

Zvolensky, M. J., Manning, K., Garey, L., Mayorga, N. A., & Peraza, N. (2019). Fatigue severity and electronic cigarette beliefs and use behavior. *Addictive Behaviors*, *97*, 1–6. https://doi.org/10.1016/j.addbeh.2019.05.014

Zvolensky, M. J., Mayorga, N. A., & Garey, L. (2019a). Main and interactive effects of e-cigarette use health literacy and anxiety sensitivity in terms of e-cigarette perceptions and dependence. *Cognitive Therapy and Research*, *43*(1), Article 1. APA PsycInfo®. https://doi.org/10.1007/s10608-018-9953-2

Zvolensky, M. J., Mayorga, N. A., & Garey, L. (2019b). Positive expectancies for e-cigarette use and anxiety sensitivity among adults. *Nicotine & Tobacco Research*, *21*(10), 1355–1362. https://doi.org/10.1093/ntr/nty106

Zvolensky, M. J., Shepherd, J. M., Garey, L., Case, K., & Gallagher, M. W. (2020). The influence of neuroticism in terms of E-cigarette dependence and beliefs about use and quitting among dual users of combustible and electronic cigarettes. *Addictive Behaviors*, *107*, 106396. https://doi.org/10.1016/j.addbeh.2020.106396
