# Supplementary material for: Cravings, Control, and Cessation: A Scoping Review of Perceptions of Nicotine Addiction
Source: Curr Addict Rep. 2025 Jul 11;12(1):66. doi: 10.1007/s40429-025-00673-4 (PMC12254085; doi:10.1007/s40429-025-00673-4)
Supplement: Supplementary file 2 — Supplementary file2 (DOCX 97 KB) [file 40429_2025_673_MOESM2_ESM.docx]

**Supplemental Material 2**. Item Placement Flowchart
