# Supplementary material for: Cravings, Control, and Cessation: A Scoping Review of Perceptions of Nicotine Addiction
Source: Curr Addict Rep. 2025 Jul 11;12(1):66. doi: 10.1007/s40429-025-00673-4 (PMC12254085; doi:10.1007/s40429-025-00673-4)
Supplement: Supplementary file 3 — Supplementary file3 (DOCX 26.4 KB) [file 40429_2025_673_MOESM3_ESM.docx]

**Supplemental Material 3. Remaining References**

151. Waters EA, Janssen E, Kaufman AR, Peterson LM, Muscanell NL, Guadagno RE, et al. The relationship between young adult smokers’ beliefs about nicotine addiction and smoking-related affect and cognitions. J Cancer Educ. 2016;31:338–47.

152. Alam MM, Ward KD, Bahelah R, Kalan ME, Asfar T, Eissenberg T, et al. The Syrian Center for Tobacco Studies-13 (SCTS-13): Psychometric evaluation of a waterpipe-specific nicotine dependence instrument. Drug Alcohol Depend. 2020;215:108192.

153. Balmford J, Borland R. What does it mean to want to quit? Drug Alcohol Rev. 2008;27:21–7.

154. Hughes JR, Naud S. Perceived role of motivation and self-efficacy in smoking cessation: A secondary data analysis. Addict Behav. 2016;61:58–61.

155. Berg MB, Lin L, White M, Alfonso-Barry J. Attitudinal and behavioral differences between cigarette users who do and do not identify as “smokers.” J Am Coll Health. 2017;65:372–9.

156. Chaiton M, Cohen JE, Bondy SJ, Selby P, Brown KS, Ferrence R, et al. Perceived addiction as a predictor of smoking cessation among occasional smokers. Journal of Smoking Cessation. 2017;12:165–72.

157. Martin DS. Physical dependence and attributions of addiction among cigarette smokers. Addict Behav. 1990;15:69–72.

158. Sabogal F, Otero-Sabogal R, Pérez-Stable EJ, Marín BV, Marin G. Perceived self-efficacy to avoid cigarette smoking and addiction: Differences between Hispanics and non-Hispanic Whites. Hispanic Journal of Behavioral Sciences. 1989;11:136–47.

159. Yunus M, Khan Z. A baseline study of tobacco use among the staff of Aligarh Muslim University, Aligarh, India. J R Soc Health. 1997;117:359–65.

160. Ashley MJ, Cohen J, Ferrence R. “Light” and “mild” cigarettes: Who smokes them? Are they being misled? Can J Public Health. 2001;92:407–11.

161. Cooper TV, Taylor T, Murray A, DeBon MW, Vander Weg MW, Klesges RC, et al. Differences between intermittent and light daily smokers in a population of U.S. military recruits. Nicotine Tob Res. 2010;12:465–73.

162. Edwards SA, Bondy SJ, Kowgier M, McDonald PW, Cohen, Joanna E. Are occasional smokers a heterogeneous group? An exploratory study. Nicotine & Tobacco Research. 2010;12:1195–202.

163. Hughes JR, Naud S. Abstinence expectancies and quit attempts. Addictive Behaviors. 2016;63:93–6.

164. Lenk KM, Chen V, Bernat DH, Forster JL, Rode PA. Characterizing and comparing young adult intermittent and daily smokers. Subst Use Misuse. 2009;44:2128–40.

165. Li VC, Hu JH, Zhou ML, Zheng JB. Behavioral aspects of cigarette smoking among industrial college men of Shanghai, China. Am J Public Health. 1988;78:1550–3.

166. Lyna P, McBride C, Samsa G, Pollak KI. Exploring the association between perceived risks of smoking and benefits to quitting: Who does not see the link? Addict Behav. 2002;27:293–307.

167. Marin BV, Perez-Stable EJ, Marin G, Sabogal F, Otero-Sabogal R. Attitudes and behaviors of Hispanic smokers: Implications for cessation interventions. Health Education Quarterly. 1990;17:287–97.

168. Sendzik T, McDonald PW, Brown KS, Hammond D, Ferrence R. Planned quit attempts among Ontario smokers: Impact on abstinence. Addiction. 2011;106:2005–13.

169. Torchalla I, Okoli CTC, Malchy L, Johnson JL. Nicotine dependence and gender differences in smokers accessing community mental health services: Gender differences in smoking among individuals with SPMI. Journal of Psychiatric and Mental Health Nursing. 2011;18:349–58.

170. Etter J-F. Explaining the effects of electronic cigarettes on craving for tobacco in recent quitters. Drug Alcohol Depend. 2015;148:102–8.

171. Etter J-F. Throat hit in users of the electronic cigarette: An exploratory study. Psychol Addict Behav. 2016;30:93–100.

172. Maziak W, Ward KD, Eissenberg T. Factors related to frequency of narghile (waterpipe) use: The first insights on tobacco dependence in narghile users. Drug Alcohol Depend. 2004;76:101–6.

173. Schippers GM, Cox WM. Problem perception and addictive behaviors among Dutch and American college students. Drugs: Education, Prevention & Policy. 1994;1:27–35.

174. Yates EA, Dubray J, Schwartz R, Kirst M, Lacombe-Duncan A, Suwal J, et al. Patterns of cigarillo use among Canadian young adults in two urban settings. Can J Public Health. 2014;105:e11-14.

175. Camara-Medeiros A, Diemert L, O’Connor S, Schwartz R, Eissenberg T, Cohen JE. Perceived addiction to vaping among youth and young adult regular vapers. Tob Control. 2021;30:273–8.

176. Chen H-L. Consumer risk perception and addictive consumption behavior. Social Behavior and Personality: An International Journal. 2009;37:767–80.

177. DiFranza JR, Morello P, Gershenson B. The retest reliability of nicotine dependence measures. Addiction Research & Theory. 2012;20:55–63.

178. Okoli CTC, Rayens MK, Wiggins AT, Ickes MJ, Butler KM, Hahn EJ. Secondhand tobacco smoke exposure and susceptibility to smoking, perceived addiction, and psychobehavioral symptoms among college students. J Am Coll Health. 2016;64:96–103.

179. Eiser JR, Van der Pligt J, Raw M, Sutton SR. Trying to stop smoking: Effects of perceived addiction, attributions for failure, and expectancy of success. Journal of Behavioral Medicine. 1985;8:321–41.

180. Eiser JR, Van der Pligt J. “Sick” or “hooked”: smokers’ perceptions of their addiction. Addict Behav. 1986;11:11–5.

181. Farrimond H. A typology of vaping: Identifying differing beliefs, motivations for use, identity and political interest amongst e-cigarette users. International Journal of Drug Policy. 2017;48:81–90.

182. Gillies PA, Madeley RJ, Power FL. Why do pregnant women smoke? Public Health. 1989;103:337–43.

183. Gilpin EA, Emery S, White MM, Pierce JP. Does tobacco industry marketing of “light” cigarettes give smokers a rationale for postponing quitting? Nicotine Tob Res. 2002;4 Suppl 2:S147-155.

184. Palinkas LA, Pierce J, Rosbrook BP, Pickwell S, Johnson M, Bal DG. Cigarette smoking behavior and beliefs of Hispanics in California. American Journal of Preventive Medicine. 1993;9:331–7.

185. Levinson AH, Campo S, Gascoigne J, Jolly O, Zakharyan A, Tran ZV. Smoking, but not smokers: Identity among college students who smoke cigarettes. Nicotine Tob Res. 2007;9:845–52.

186. Perski O, Herd N, West R, Brown J. Perceived addiction to smoking and associations with motivation to stop, quit attempts and quitting success: A prospective study of English smokers. Addict Behav. 2019;90:306–11.

187. Vander Martin R, Cummings SR, Coates TJ. Ethnicity and smoking: Differences in White, Black, Hispanic, and Asian medical patients who smoke. Am J Prev Med. 1990;6:194–9.

188. Zinser MC, Pampel FC, Flores E. Distinct beliefs, attitudes, and experiences of Latino smokers: relevance for cessation interventions. Am J Health Promot. 2011;25:eS1-15.

189. Stippekohl B, Winkler MH, Walter B, Kagerer S, Mucha RF, Pauli P, et al. Neural responses to smoking stimuli are influenced by smokers’ attitudes towards their own smoking behavior. PLoS ONE. 2012;7:e46782.

190. Goniewicz ML, Lingas EO, Hajek P. Patterns of electronic cigarette use and user beliefs about their safety and benefits: an internet survey. Drug Alcohol Rev. 2013;32:133–40.

191. Thawal VP, Tzelepis F, Ahmadi S, Palazzi K, Paul C. Addiction perceptions among users of smokeless or combustible tobacco attending a tertiary care hospital in India. Drug and Alcohol Review. 2022;41:1184–94.

192. Konkolÿ Thege B, Colman I, El-guebaly N, Hodgins DC, Patten SB, Schopflocher D, et al. Substance-related and behavioural addiction problems: Two surveys of Canadian adults. Addiction Research & Theory. 2015;23:34–42.

193. United States Department of Health and Human Services, National Institutes of Health, National Institute on Drug Abuse, Food and Drug Administration, Center for Tobacco Products. Population Assessment of Tobacco and Health (PATH) Study [United States] Public-Use Files. Ann Arbor, MI: Inter-university Consortium for Political and Social Research [distributor]; 2021.

194. Mostafa A. Self-reported addiction to and perceived behavioural control of waterpipe tobacco smoking and its patterns in Egypt: policy implications. East Mediterr Health J. 2020;26:18–28.

195. Weinstein ND, Slovic P, Gibson G. Accuracy and optimism in smokers’ beliefs about quitting. Nicotine Tob Res. 2004;6 Suppl 3:S375-380.

196. Vu T-HT, Hart JL, Groom A, Landry RL, Walker KL, Giachello AL, et al. Age differences in electronic nicotine delivery systems (ENDS) usage motivations and behaviors, perceived health benefit, and intention to quit. Addict Behav. 2019;98:106054.

197. Alizaga NM, Hartman-Filson M, Elser H, Halpern-Felsher B, Vijayaraghavan M. Alternative flavored and unflavored tobacco product use and cigarette quit attempts among current smokers experiencing homelessness. Addictive Behaviors Reports. 2020;12:8.

198. Berg CJ, Stratton E, Schauer GL, Lewis M, Wang Y, Windle M, et al. Perceived harm, addictiveness, and social acceptability of tobacco products and marijuana among young adults: marijuana, hookah, and electronic cigarettes win. Subst Use Misuse. 2015;50:79–89.

199. Berg CJ, Haardörfer R, Wagener TL, Kegler MC, Windle M. Correlates of allowing alternative tobacco product or marijuana use in the homes of young adults. Pediatrics. 2018;141:S10–20.

200. Berg CJ, Romm KF, Patterson B, Wysota CN. Heated tobacco product awareness, use, and perceptions in a sample of young adults in the United States. Nicotine Tob Res. 2021;23:1967–71.

201. Jiang N, Cleland CM, Wang MP, Kwong A, Lai V, Lam TH. Perceptions and use of e-cigarettes among young adults in Hong Kong. BMC Public Health. 2019;19:1123.

202. Perks SN, Haardörfer R, Windle M, Berg CJ. Tobacco abstinence motives in young adult college students: Scale development and validation. Am J Health Behav. 2019;43:464–77.

203. Blomqvist J. Perceptions of addiction and recovery in Sweden: The influence of respondent characteristics. Addiction Research & Theory. 2012;20:435–46.

204. Calhoun JF. Attitudes toward the sale and use of drugs —A cross-sectional analysis of those who used drugs. J Youth Adolescence. 1974;3:31–47.

205. Case KR, Hinds JT, Creamer MR, Loukas A, Perry CL. Who is JUULing and why? An examination of young adult electronic nicotine delivery systems users. J Adolesc Health. 2020;66:48–55.

206. Cooper M, Loukas A, Harrell MB, Perry CL. College students’ perceptions of risk and addictiveness of e-cigarettes and cigarettes. J Am Coll Health. 2017;65:103–11.

207. Cooper M, Loukas A, Case KR, Marti CN, Perry CL. A longitudinal study of risk perceptions and e-cigarette initiation among college students: Interactions with smoking status. Drug Alcohol Depend. 2018;186:257–63.

208. North C, Li X, Grossberg LA, Loukas A. A one year prospective examination of risk factors for pod-vape use among young adults. Drug Alcohol Depend. 2021;229:109141.

209. Creamer MR, Loukas A, Li X, Pasch KE, Case K, Crook B, et al. College students’ perceptions and knowledge of hookah use. Drug Alcohol Depend. 2016;168:191–5.

210. Jongenelis MI, Kameron C, Rudaizky D, Slevin T, Pettigrew S. Perceptions of the harm, addictiveness, and smoking cessation effectiveness of e-cigarettes among Australian young adults. Addict Behav. 2019;90:217–21.

211. Kimber C, Frings D, Cox S, Albery I, Dawkins L. The effects of the European e-cigarette health warnings and comparative health messages on non-smokers’ and smokers’ risk perceptions and behavioural intentions. BMC Public Health. 2018;18:1259.

212. Kimber C, Frings D, Cox S, Albery IP, Dawkins L. Communicating the relative health risks of E-cigarettes: An online experimental study exploring the effects of a comparative health message versus the EU nicotine addiction warnings on smokers’ and non-smokers’ risk perceptions and behavioural intentions. Addict Behav. 2020;101:106177.

213. Kolar SK, Rogers BG, Hooper MW. Support for indoor bans on electronic cigarettes among current and former smokers. Int J Environ Res Public Health. 2014;11:12174–89.

214. Mays D, Smith C, Johnson AC, Tercyak KP, Niaura RS. An experimental study of the effects of electronic cigarette warnings on young adult nonsmokers’ perceptions and behavioral intentions. Tob Induced Dis. 2016;14:17.

215. Vogel EA, Henriksen L, Schleicher NC, Prochaska JJ. Young people’s e-cigarette risk perceptions, policy attitudes, and past-month nicotine vaping in 30 U.S. cities. Drug Alcohol Depend. 2021;229:109122.

216. Webb Hooper M, Kolar SK. Racial/ethnic differences in electronic cigarette knowledge, social norms, and risk perceptions among current and former smokers. Addict Behav. 2017;67:86–91.

217. Havermans A, Pennings JLA, Hegger I, Elling JM, de Vries H, Pauwels CGGM, et al. Awareness, use and perceptions of cigarillos, heated tobacco products and nicotine pouches: A survey among Dutch adolescents and adults. Drug Alcohol Depend. 2021;229:109136.

218. Center for Disease Control and Prevention. National Adult Tobacco Survey [Internet]. 2015. Available from: https://www.cdc.gov/tobacco/data_statistics/surveys/nats/pdfs/2013-2014-questionnaire-tag508.pdf

219. Arnett JJ. Optimistic bias in adolescent and adult smokers and nonsmokers. Addict Behav. 2000;25:625–32.

220. Ashley MJ, Cohen J, Bull S, Ferrence R, Poland B, Pederson L, et al. Knowledge about tobacco and attitudes toward tobacco control: How different are smokers and nonsmokers? Can J Public Health. 2000;91:376–80.

221. Chassin L, Presson CC, Rose J, Sherman SJ. What is addiction? Age-related differences in the meaning of addiction. Drug and Alcohol Dependence. 2007;87:30–8.

222. Konkolÿ Thege B, Colman I, el-Guebaly N, Hodgins DC, Patten SB, Schopflocher D, et al. Social judgments of behavioral versus substance-related addictions: A population-based study. Addict Behav. 2015;42:24–31.

223. Cunningham JA. Smokers and non-smokers differ in their beliefs about their addiction: Public health implications. Canadian Journal of Public Health = Revue Canadienne De Sante Publique. 2012;103.

224. Eiser JR, Sutton SR, Wober M. Smokers, non-smokers and the attribution of addiction. Br J Soc Clin Psychol. 1977;16:329–36.

225. Sadava SW, Weithe H. Maintenance and attributions about smoking among smokers, nonsmokers, and ex-smokers. International Journal of the Addictions. 1985;20:1533–44.

226. Smith P, Bansal-Travers M, O’Connor R, Brown A, Banthin C, Guardino-Colket S, et al. Correcting over 50 years of tobacco industry misinformation. Am J Prev Med. 2011;40:690–8.

227. Vigna-Taglianti F, Alesina M, Damjanović L, Mehanović E, Akanidomo I, Pwajok J, et al. Knowledge, attitudes and behaviours on tobacco, alcohol and other drugs among Nigerian secondary school students: Differences by geopolitical zones. Drug Alcohol Rev. 2019;38:712–24.

228. Yel D, Bui A, Job JS, Knutsen S, Singh PN. Beliefs about tobacco, health, and addiction among adults in Cambodia: Findings from a national survey. J Relig Health. 2013;52:904–14.

229. Majeed BA, Weaver SR, Gregory KR, Whitney CF, Slovic P, Pechacek TF, et al. Changing perceptions of harm of e-cigarettes among U.S. adults, 2012-2015. Am J Prev Med. 2017;52:331–8.

230. Meijer E, Chavannes NH. Lacking willpower? A latent class analysis of healthcare providers’ perceptions of smokers’ responsibility for smoking. Patient Education and Counseling. 2021;104:620–6.

231. Rahman MA, Mahmood MA, Spurrier N, Rahman M, Choudhury SR, Leeder S. Why do Bangladeshi people use smokeless tobacco products? Asia Pac J Public Health. 2015;27:NP2197-2209.

232. Wackowski OA, Sontag JM, Hammond D, O’Connor RJ, Ohman-Strickland PA, Strasser AA, et al. The impact of e-cigarette warnings, warning themes and inclusion of relative harm statements on young adults’ e-cigarette perceptions and use intentions. Int J Environ Res Public Health. 2019;16:E184.

233. Al-Naggar RA, Bobryshev YV, Anil S. Pattern of shisha and cigarette smoking in the general population in Malaysia. Asian Pac J Cancer Prev. 2014;15:10841–6.

234. Al-Naggar RA, Saghir FSA. Water pipe (shisha) smoking and associated factors among Malaysian university students. Asian Pac J Cancer Prev. 2011;12:3041–7.

235. Asfar T, Ward KD, Eissenberg T, Maziak W. Comparison of patterns of use, beliefs, and attitudes related to waterpipe between beginning and established smokers. BMC Public Health. 2005;5:19.

236. Aqeeli AA, Makeen AM, Al Bahhawi T, Ryani MA, Bahri AA, Alqassim AY, et al. Awareness, knowledge and perception of electronic cigarettes among undergraduate students in Jazan Region, Saudi Arabia. Health Soc Care Community. 2022;30:706–13.

237. Choi K, Bestrashniy J, Forster J. Trends in awareness, use of, and beliefs about electronic cigarette and snus among a longitudinal cohort of US midwest young adults. Nicotine Tob Res. 2018;20:239–45.

238. Choi K, Forster JL. Beliefs and experimentation with electronic cigarettes: a prospective analysis among young adults. Am J Prev Med. 2014;46:175–8.

239. Correa JB, Brandon KO, Meltzer LR, Hoehn HJ, Piñeiro B, Brandon TH, et al. Electronic cigarette use among patients with cancer: Reasons for use, beliefs, and patient-provider communication. Psychooncology. 2018;27:1757–64.

240. Hershberger AR, Karyadi KA, VanderVeen JD, Cyders MA. Beliefs about the direct comparison of e-cigarettes and cigarettes. Substance Use & Misuse. 2017;52:982–91.

241. Lee H-Y, Lin H-C, Seo D-C, Lohrmann DK. The effect of e-cigarette warning labels on college students’ perception of e-cigarettes and intention to use e-cigarettes. Addict Behav. 2018;76:106–12.

242. Melin K, Conte-Schmidt N, Martínez-Arroyo K, Rosa-Pérez K, Soto-Avilés AE, Hernández-Muñoz JJ. Knowledge and perceptions of e-cigarettes and the motivations for their use: Talking to smokers (e-cigarettes and/or conventional cigarettes) and non-smokers in Puerto Rico. P R Health Sci J. 2018;37:148–54.

243. Nicksic NE, Snell LM, Rudy AK, Cobb CO, Barnes AJ. Tobacco marketing, e-cigarette susceptibility, and perceptions among adults. Am J Health Behav. 2017;41:579–90.

244. Peters EN, Harrell PT, Hendricks PS, O’Grady KE, Pickworth WB, Vocci FJ. Electronic cigarettes in adults in outpatient substance use treatment: Awareness, perceptions, use, and reasons for use. Am J Addict. 2015;24:233–9.

245. Pokhrel P, Fagan P, Herzog TA, Chen Q, Muranaka N, Kehl L, et al. E-cigarette advertising exposure and implicit attitudes among young adult non-smokers. Drug Alcohol Depend. 2016;163:134–40.

246. Stein MD, Caviness CM, Grimone K, Audet D, Borges A, Anderson BJ. E-cigarette knowledge, attitudes, and use in opioid dependent smokers. Journal of Substance Abuse Treatment. 2015;52:73–7.

247. Trumbo CW. Influence of risk perception on attitudes and norms regarding electronic cigarettes. Risk Analysis. 2018;38:906–16.

248. Borgan SM, Marhoon ZA, Whitford DL. Beliefs and perceptions toward quitting waterpipe smoking among cafe waterpipe tobacco smokers in Bahrain. Nicotine Tob Res. 2013;15:1816–21.

249. Ward KD, Hammal F, VanderWeg MW, Eissenberg T, Asfar T, Rastam S, et al. Are waterpipe users interested in quitting? Nicotine & Tobacco Research. 2005;7:149–56.

250. Brennan E, Gibson L, Momjian A, Hornik RC. Are young people’s beliefs about menthol cigarettes associated with smoking-related intentions and behaviors? Nicotine Tob Res. 2015;17:81–90.

251. Byron MJ, Hall MG, King JL, Ribisl KM, Brewer NT. Reducing nicotine without misleading the public: Descriptions of cigarette nicotine level and accuracy of perceptions about nicotine content, addictiveness, and risk. Nicotine Tob Res. 2019;21:S101–7.

252. Morgan JC, Cappella JN. Harm perceptions and beliefs about potential modified risk tobacco products. Int J Environ Res Public Health. 2021;18:E576.

253. Choi K, Forster J. Awareness, perceptions and use of snus among young adults from the upper Midwest region of the USA. Tob Control. 2013;22:412–7.

254. Cummings KM, Hyland A, Bansal MA, Giovino GA. What do Marlboro Lights smokers know about low-tar cigarettes? Nicotine Tob Res. 2004;6 Suppl 3:S323-332.

255. Rass O, Pacek LR, Johnson PS, Johnson MW. Characterizing use patterns and perceptions of relative harm in dual users of electronic and tobacco cigarettes. Exp Clin Psychopharmacol. 2015;23:494–503.

256. Egnot E, Jordan K, Elliott JO. Associations with resident physicians’ early adoption of electronic cigarettes for smoking cessation. Postgrad Med J. 2017;93:319–25.

257. Rahman MA, Joseph B, Nimmi N. Electronic cigarettes or vaping: Are there any differences in the profiles, use and perceptions between a developed and a developing country? Int J Environ Res Public Health. 2022;19:1673.

258. Hefner KR, Sollazzo A, Mullaney S, Coker KL, Sofuoglu M. E-cigarettes, alcohol use, and mental health: Use and perceptions of e-cigarettes among college students, by alcohol use and mental health status. Addict Behav. 2019;91:12–20.

259. Heinz AJ, Giedgowd GE, Crane NA, Veilleux JC, Conrad M, Braun AR, et al. A comprehensive examination of hookah smoking in college students: Use patterns and contexts, social norms and attitudes, harm perception, psychological correlates and co-occurring substance use. Addict Behav. 2013;38:2751–60.

260. Hughes JR, Keely JP, Callas PW. Ever users versus never users of a “less risky” cigarette. Psychol Addict Behav. 2005;19:439–42.

261. Shadel WG, Lerman C, Cappella J, Strasser AA, Pinto A, Hornik R. Evaluating smokers’ reactions to advertising for new lower nicotine quest cigarettes. Psychol Addict Behav. 2006;20:80–4.

262. Noonan D, Patrick ME. Factors associated with perceptions of hookah addictiveness and harmfulness among young adults. Substance Abuse. 2013;34:83–5.

263. Smith-Simone S, Maziak W, Ward KD, Eissenberg T. Waterpipe tobacco smoking: Knowledge, attitudes, beliefs, and behavior in two U.S. samples. Nicotine & Tobacco Research. 2008;10:393–8.

264. Lund I, Scheffels J. Perceptions of relative risk of disease and addiction from cigarettes and snus. Psychol Addict Behav. 2014;28:367–75.

265. Mays D, Moran MB, Levy DT, Niaura RS. The impact of health warning labels for Swedish snus advertisements on young adults’ snus perceptions and behavioral intentions. Nicotine Tob Res. 2016;18:1371–5.

266. Mercincavage M, Lochbuehler K, Villanti AC, Wileyto EP, Audrain-McGovern J, Strasser AA. Examining risk perceptions among daily smokers naïve to reduced nicotine content cigarettes. Nicotine Tob Res. 2019;21:985–90.

267. Moysidou A, Farsalinos KE, Voudris V, Merakou K, Kourea K, Barbouni A. Knowledge and perceptions about nicotine, nicotine replacement therapies and electronic cigarettes among healthcare professionals in Greece. Int J Environ Res Public Health. 2016;13:E514.

268. Perman-Howe PR, Horton M, Robson D, McDermott MS, McNeill A, Brose LS. Harm perceptions of nicotine-containing products and associated sources of information in UK adults with and without mental ill health: A cross-sectional survey. Addiction. 2022;117:715–29.

269. Wilson S, Partos T, McNeill A, Brose LS. Harm perceptions of e-cigarettes and other nicotine products in a UK sample. Addiction. 2019;114:879–88.

270. Primack BA, Sidani J, Agarwal AA, Shadel WG, Donny EC, Eissenberg TE. Prevalence of and associations with waterpipe tobacco smoking among U.S. university students. Ann Behav Med. 2008;36:81–6.

271. Queloz S, Etter J-F. A survey of users of the IQOS tobacco vaporizer: perceived dependence and perceived effects on cigarette withdrawal symptoms. J Addict Dis. 2021;39:208–14.

272. Salih S, Shaban S, Athwani Z, Alyahyawi F, Alharbi S, Ageeli F, et al. Prevalence, predictors, and characteristics of waterpipe smoking among Jazan University students in Saudi Arabia: A cross-sectional study. Ann Glob Health. 2020;86:87.

273. Saravanan C, Attlee A, Sulaiman N. A cross sectional study on knowledge, beliefs and psychosocial predictors of shisha smoking among university students in Sharjah, United Arab Emirates. Asian Pac J Cancer Prev. 2019;20:903–9.

274. Ward KD, Eissenberg T, Gray JN, Srinivas V, Wilson N, Maziak W. Characteristics of U.S. waterpipe users: a preliminary report. Nicotine Tob Res. 2007;9:1339–46.

275. Grassi MC, Chiamulera C, Baraldo M, Culasso F, Ferketich AK, Raupach T, et al. Cigarette smoking knowledge and perceptions among students in four Italian medical schools. Nicotine Tob Res. 2012;14:1065–72.

276. Yan J, Xiao S, Ouyang D, Jiang D, He C, Yi S. Smoking behavior, knowledge, attitudes and practice among health care providers in Changsha City, China. Nicotine Tob Res. 2008;10:737–44.

277. Cano S, Chrea C, Salzberger T, Alfieri T, Emilien G, Mainy N, et al. Development and validation of a new instrument to measure perceived risks associated with the use of tobacco and nicotine-containing products. Health Qual Life Outcomes. 2018;16:192.

278. Pokhrel P, Little MA, Fagan P, Muranaka N, Herzog TA. Electronic cigarette use outcome expectancies among college students. Addict Behav. 2014;39:1062–5.

279. Pokhrel P, Fagan P, Herzog TA, Laestadius L, Buente W, Kawamoto CT, et al. Social media e-cigarette exposure and e-cigarette expectancies and use among young adults. Addictive Behaviors. 2018;78:51–8.

280. Pokhrel P, Lam TH, Pagano I, Kawamoto CT, Herzog TA. Young adult e-cigarette use outcome expectancies: Validity of a revised scale and a short scale. Addictive Behaviors. 2018;78:193–9.

281. Selekoğlu Ok Y, Bektas M, Pokhrel P. Psychometric properties of Turkish version of the E-cigarette Use Outcome Expectancies Scale. J Addict Nurs. 2020;31:124–33.

282. CDC. Why quitting smoking is hard [Internet]. 2022. Available from: https://www.cdc.gov/tobacco/campaign/tips/quit-smoking/quit-smoking-medications/why-quitting-smoking-is-hard/index.html

283. Fitz CC, Kaufman A, Moore PJ. Lay theories of smoking and young adult nonsmokers’ and smokers’ smoking expectations. J Health Psychol. 2015;20:438–45.

284. Vietor N. Understanding smoking relapse from an implicit theories framework. University of Houston; 2001.

285. McKelvey K, Halpern-Felsher B. How and why California young adults are using different brands of pod-type electronic cigarettes in 2019: Implications for researchers and regulators. Journal of Adolescent Health. 2020;67:46–52.

286. Wipfli HL, Berman M, Hanson K, Kelder S, Solis A, Villanti AC, et al. Defining tobacco regulatory science competencies. NICTOB. 2017;19:222–30.
